# Combination of X-ray crystallography, SAXS and DEER to obtain the structure of the FnIII-3,4 domains of integrin α6β4

**DOI:** 10.1107/S1399004715002485

**Published:** 2015-03-27

**Authors:** Noelia Alonso-García, Inés García-Rubio, José A. Manso, Rubén M. Buey, Hector Urien, Arnoud Sonnenberg, Gunnar Jeschke, José M. de Pereda

**Affiliations:** a Instituto de Biología Molecular y Celular del Cancer, Consejo Superior de Investigaciones Científicas – University of Salamanca, Campus Unamuno, 37007 Salamanca, Spain; b Laboratory of Physical Chemistry, ETH Zürich, Vladimir-Prelog-Weg 2, CH-8093 Zürich, Switzerland; c Centro Universitario de la Defensa, Academia General Militar, Carretera de Huesca s/n, 50090 Zaragoza, Spain; d Metabolic Engineering Group, Department of Microbiology and Genetics, University of Salamanca, Campus Unamuno, 37007 Salamanca, Spain; e Netherlands Cancer Institute, Plesmanlaan 121, 1066 CX Amsterdam, The Netherlands

**Keywords:** integrin α6β4, FnIII-3, FnIII-4, small-angle X-ray scattering, double electron–electron resonance

## Abstract

Integrin α6β4 is a major component of hemidesmosomes that mediate the stable anchorage of epithelial cells to the underlying basement membrane. Integrin α6β4 has also been implicated in cell proliferation and migration and in carcinoma progression. The third and fourth fibronectin type III domains (FnIII-3,4) of integrin β4 mediate binding to the hemidesmosomal proteins BPAG1e and BPAG2, and participate in signalling. Here, it is demonstrated that X-ray crystallography, small-angle X-ray scattering and double electron–electron resonance (DEER) complement each other to solve the structure of the FnIII-3,4 region. The crystal structures of the individual FnIII-3 and FnIII-4 domains were solved and the relative arrangement of the FnIII domains was elucidated by combining DEER with site-directed spin labelling. Multiple structures of the interdomain linker were modelled by Monte Carlo methods complying with DEER constraints, and the final structures were selected against experimental scattering data. FnIII-3,4 has a compact and cambered flat structure with an evolutionary conserved surface that is likely to correspond to a protein-interaction site. Finally, this hybrid method is of general application for the study of other macromolecules and complexes.

## Abstract 2

The structure of the FnIII-3,4 region of integrin β4 was solved using a hybrid approach that combines crystallographic structures, SAXS, DEER and molecular modelling. The structure helps in understanding how integrin β4 might bind to other hemidesmosomal proteins and mediate signalling.

## 1. Introduction   

Integrins are αβ heterodimeric cell-surface adhesion and signalling receptors. In vertebrates, there are 24 receptors formed by combinations of 18 α and eight β subunits (Barczyk *et al.*, 2010 ▶; Hynes, 2002 ▶). The α and β subunits have large N-terminal extracellular domains, a single transmembrane segment and C-terminal cytoplasmic tails that interact with cytoskeletal and signalling proteins (Campbell & Humphries, 2011 ▶).

The β4 subunit only pairs with the α6 subunit to form the α6β4 integrin, which is a laminin receptor required for maintaining the integrity of epithelia. Loss of α6β4 results in the blistering disease junctional epidermolysis bullosa associated with pyloric atresia (JEB-PA), which is characterized by fragility of the skin (Chung & Uitto, 2010 ▶). α6β4 also participates in signalling pathways involved in keratinocyte proliferation and migration, and in carcinoma invasion and survival (Wilhelmsen *et al.*, 2006 ▶).

α6β4 plays a major role in cell adhesion as an essential component of hemidesmosomes (HDs), junctional complexes that provide firm attachment of the basal layer of epithelial cells to the basement membrane by connecting the extracellular matrix to the intermediate filaments (de Pereda, Ortega *et al.*, 2009 ▶; Margadant *et al.*, 2008 ▶). In (pseudo) stratified epithelia the HDs contain α6β4, the bullous pemphigoid antigen 2 (BPAG2, also known as BP180 or collagen XVII), the tetraspanin CD151 and two members of the plakin family of cytolinkers: plectin and BPAG1e (BP230). α6β4 binds to CD151, BPAG2, plectin and BPAG1e, acting as a hub for the assembly, organization and regulation of HDs (Margadant *et al.*, 2008 ▶).

**Figure 1:**
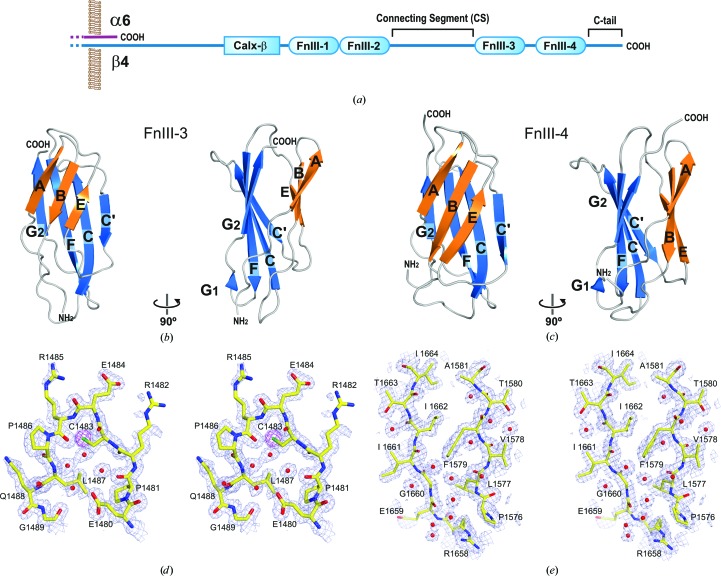
Crystal structures of the FnIII-3 and FnIII-4 domains of integrin β4. (*a*) Diagram of the domain structure of the α6β4 cytoplasmic region. (*b*, *c*) Orthogonal views of ribbon representations of the crystal structures of the FnIII-3 (*b*) and FnIII-4 (*c*) domains. (*d*, *e*) Stereoviews of 2*mF*_obs_ − *DF*_calc_ simulated-annealing OMIT maps (contoured at 1σ) of representative regions of FnIII-3 (*d*) and FnIII-4 (*e*) superimposed on the refined models. For FnIII-3, an anomalous difference map (contoured at 4σ) is shown in magenta. Phases were calculated from models in which the residues and the water molecules shown were removed and the *B* factors were reset to the Wilson-plot value; the models were then refined by simulated annealing (start temperature 4000 K).

The β4 subunit mediates most of the intracellular inter­actions of α6β4. Mice carrying a deletion of the β4 cytodomain had severe skin defects similar to those observed in the human disease JEB-PA and failed to form HDs (Murgia *et al.*, 1998 ▶). The cytoplasmic moiety of β4 (∼1000 residues) is uniquely large among the integrin family. It has a modular structure that contains a Calx-β domain (Alonso-García *et al.*, 2009 ▶) followed by four fibronectin type III (FnIII) domains arranged in two pairs (FnIII-1,2 and FnIII-3,4; Fig. 1 ▶ *a*). FnIII-2 and FnIII-3 are separated by a region of ∼140 residues named the connecting segment (CS). Finally, an 86-residue C-terminal tail (C-tail) extends downstream of FnIII-4.

The FnIII domains of β4 participate in protein–protein interactions in the HDs. FnIII-1,2 and part of the CS bind to the actin-binding domain of plectin (de Pereda, Ortega *et al.*, 2009 ▶; Geerts *et al.*, 1999 ▶). The C-terminal part of the CS, FnIII-4 and the C-tail bind to the plakin domain of plectin (Frijns *et al.*, 2012 ▶; Koster *et al.*, 2004 ▶; Rezniczek *et al.*, 1998 ▶). FnIII-3,4 together with the final sequence of the CS interact with BPAG1e, and FnIII-3 binds to BPAG2 (Koster *et al.*, 2003 ▶). FnIII-3 has also been implicated in cellular signalling. Y1494 is required for the activation of phosphatidylinositol 3-kinase and stimulation of invasion after ligation of α6β4 (Shaw, 2001 ▶). Phosphorylation of Y1494, together with Y1257 in FnIII-2 and Y1440 in the CS, results in binding of the phosphatase Shp2 to β4 and stimulation of the Erk pathway (Bertotti *et al.*, 2006 ▶). α6β4 is also coupled *via* Shc to Erk through phosphorylation of Y1526 in FnIII-3 (Dans *et al.*, 2001 ▶).

Yeast two-hybrid and blot-overlay experiments using β4 fragments suggest that the CS and the C-tail interact with each other (Koster *et al.*, 2004 ▶; Rezniczek *et al.*, 1998 ▶). Recently, it has been shown that the C-tail is in close proximity to the CS of the same molecule in keratinocytes, suggesting that β4 adopts a folded-back structure in which the linker between FnIII-3 and FnIII-4 might allow a bent conformation (Frijns *et al.*, 2012 ▶).

In spite of the role of FnIII-3,4 in protein–protein interactions and in the arrangement of the β4 cytodomain, the structural organization of this region remained largely uncharacterized. Here, we have combined X-ray crystallo­graphy, small-angle X-ray scattering (SAXS) and electron paramagnetic resonance (EPR) spectroscopy to elucidate the structure of FnIII-3,4.

## 2. Materials and methods   

### 2.1. Protein expression and purification   

The cDNA sequences of human integrin β4 (UniProt P16144-2) coding for the regions 1457–1548, 1572–1666 and 1457–1666 were amplified by polymerase chain reaction using IMAGE clone 3640058 (GenBank BE737196) as a template. The amplified DNA fragments were cloned into a modified version of the pET-15b vector (Alonso-García *et al.*, 2009 ▶) using NdeI and BamHI sites that were introduced into the forward and reverse primers, respectively. Soluble proteins were produced in *Escherichia coli* strain BL21(DE3) grown in Terrific Broth medium supplemented with 100 mg l^−1^ ampicillin. Protein synthesis was induced with 0.2 m*M* isopropyl β-d-1-thiogalactopyranoside; the fragments 1457–1548 and 1457–1666 were expressed at 310 K for 3 h, while the fragment 1572–1666 was expressed at 288 K for 12 h. The proteins were purified by nickel-affinity chromatography as described in García-Alvarez *et al.* (2003 ▶). The His tag was cleaved by digestion with *Tobacco etch virus* protease, which leaves four additional residues (sequence GSHM) coded by the vector at the N-terminus. The His tag was removed by a second nickel-affinity chromatography step. Finally, the proteins were dialyzed against the desired buffer and were concentrated by ultrafiltration using Amicon cells (Merck Millipore).

### 2.2. Crystallization and structure determination of FnIII-3   

Crystals of FnIII-3 (residues 1457–1548) were grown by vapour diffusion at 277 K by mixing protein solution at 20 mg ml^−1^ in 20 m*M* Tris–HCl pH 7.5, 4 m*M* DTT with an equal volume of mother liquor consisting of 0.1 *M* Tris–HCl pH 7.0, 12.5% glycerol, 36% PEG 600, 0.5 *M* ammonium sulfate. Crystals were cooled by direct immersion into liquid N_2_. Data were collected at 120 K using an FR591 rotating-anode generator (Bruker AXS) and a MAR345 detector (MAR Research). Diffraction intensities were indexed and integrated with *XDS*, reduced with *XSCALE* and converted into structure-factor moduli with *XDSCONV* (Kabsch, 2010 ▶).

The FnIII-3 crystal belonged to space group *I*2_1_2_1_2_1_ and contained two molecules in the asymmetric unit (∼47% solvent content, Matthews coefficient 2.33 Å^3^ Da^−1^; Table 1 ▶). The structure was phased by molecular replacement using *Phaser* (McCoy *et al.*, 2007 ▶) within the *CCP*4 suite (Winn *et al.*, 2011 ▶). The structure of FnIII-2 from β4 (PDB entry 1qg3; de Pereda *et al.*, 1999 ▶) was used to build a mixed search model for molecular replacement by homology modelling with *SCWRL*4 (Krivov *et al.*, 2009 ▶). Refinement was performed against data to 1.60 Å resolution using *phenix.refine* (Afonine *et al.*, 2012 ▶) combined with manual model building using *Coot* (Emsley *et al.*, 2010 ▶). Simulated annealing was used in the initial stages of refinement. Later on, restrained positional refinement, individual isotropic *B*-factor restrained refinement, bulk-solvent correction and refinement of translation/libration/screw (TLS) parameters were used. Two TLS groups, one for each protein molecule, were refined. H atoms were included using a riding model. Two electron densities with a tetrahedral shape whose centres corresponded to peaks in native anomalous difference maps were modelled as sulfate ions. The final model contains residues 1457–1548 in each protein chain; molecules *A* and *B* contain three (SHM) and one (M) additional residues at the N-terminus, respectively, encoded by the vector. The structure also contains 219 solvent molecules and two PEG fragments. The model has excellent geometry, with 98.3 and 1.7% of the main-chain torsion angles located in the favoured and the additionally allowed regions, respectively, of the Ramachandran plot determined with *MolProbity* (Chen *et al.*, 2010 ▶).

### 2.3. Crystallization and structure determination of FnIII-4   

Crystals of FnIII-4 (residues 1572–1666) were obtained at 277 K by vapour diffusion; protein solution at 27 mg ml^−1^ in 10 m*M* Tris–HCl pH 7.5, 2 m*M* DTT was mixed with an equal volume of crystallization solution consisting of 0.1 *M* sodium acetate pH 4.2, 0.72 *M* NaH_2_PO_4_, 1.08 *M* K_2_HPO_4_, 2 m*M* DTT. Before data collection, native crystals were transferred into a cryoprotectant solution consisting of 0.1 *M* sodium acetate pH 4.2, 0.72 *M* NaH_2_PO_4_, 1.08 *M* K_2_HPO_4_, 2 m*M* DTT, 5% glycerol and were cooled by immersion in liquid N_2_. Alternatively, a crystal was derivatized with heavy atoms by soaking it for 6 h at 277 K in 0.1 *M* sodium acetate pH 4.2, 0.72 *M* NaH_2_PO_4_, 1.08 *M* K_2_HPO_4_, 1 m*M* ethylmercurithio­salicylate (EMTS). Excess EMTS was removed by a brief transfer into the cryoprotectant solution without DTT prior to immersion in liquid N_2_. Data from initial native (native 1) and EMTS-derivatized crystals were collected at 100 K using a MICROSTAR-H rotating-anode generator (Bruker AXS). Subsequently, data from a second native crystal (native 2) were collected at 100 K on the PXIII beamline of the Swiss Light Source, Villigen, Switzerland. Diffraction data were processed as for the FnIII-3 crystals.

The crystals belonged to space group *P*4_1_2_1_2 and contained a single FnIII-4 molecule in the asymmetric unit (∼59% solvent content, Matthews coefficient 2.95 Å^3^ Da^−1^; Table 1 ▶). Phases were obtained by single isomorphous replacement with anomalous scattering (SIRAS) using the native 1 and mercury-derivative data sets. Determination of the heavy-atom substructure (one high-occupancy Hg site and three minor sites), calculation of its approximate substructure amplitudes and initial phase calculations were performed with *SHELXC*/*D*/*E* (Sheldrick, 2010 ▶) and *HKL*2*MAP* (Pape & Schneider, 2004 ▶). Phase probability distributions for the major Hg site were further refined with *autoSHARP* (Vonrhein *et al.*, 2007 ▶), which allowed the confirmation of three minor Hg sites. The phases were improved and extended with *SOLOMON* (Abrahams & Leslie, 1996 ▶). A map of outstanding quality and detail was calculated using the SIRAS-derived phases (Supplementary Fig. S1), which allowed the building of 94 out of 95 residues using *ARP*/*wARP* (Langer *et al.*, 2008 ▶). Refinement was performed similarly as for the FnIII-3 structure. After initial refinement against the native 1 data to 1.80 Å resolution, the structure was refined against the native 2 data set extending to 1.50 Å resolution. The same subset of reflections, in the resolution range common to the two data sets, was used for the calculation of *R*_free_. The TLS parameters of five groups were refined. The refinement converged at *R*_work_ and *R*_free_ values of 0.195 and 0.218, respectively. The refined model includes residues 1572–1666, two additional residues (HM) encoded by the vector at the N-terminus and 122 solvent molecules. The structure has very good geometry; 98.2% of the main-chain torsion angles are in the most favoured regions of the Ramachandran plot and the remainder (1.8%) fall into additionally allowed regions.

### 2.4. Analysis of atomic structures   

Pairwise superimposition of structures was performed with *LSQKAB* (Kabsch, 1976 ▶). Simultaneous superimposition of multiple structures was performed with *THESEUS*, which applies a maximum-likelihood method (Theobald & Steindel, 2012 ▶). Electrostatic properties were calculated with *APBS* (Baker *et al.*, 2001 ▶) using the PARSE force field (Sitkoff *et al.*, 1994 ▶), and the electrostatic potentials were compared with the *webPIPSA* server (Richter *et al.*, 2008 ▶). Evolutionary conservation scores were calculated with *ConSurf* (Ashkenazy *et al.*, 2010 ▶). Molecular figures were created using *PyMOL* (v.1.5.0.2; Schrödinger).

### 2.5. SAXS measurements and analysis   

SAXS data were collected at the European Molecular Biology Laboratory (EMBL) on beamline P12 at Deutsches Elektronen Synchrotron (DESY), Hamburg, Germany using a Pilatus 2M detector (Dectris) with radiation of wavelength 1.24 Å. Proteins for SAXS analysis were additionally purified and equilibrated in 20 m*M* sodium phosphate pH 7.5, 150 m*M* NaCl, 5% glycerol, 3 m*M* DTT by size-exclusion chromatography using a HiPrep Sephacryl S300 26/60 column (GE Healthcare). Data for wild-type FnIII-3,4 were collected at a sample-to-detector distance of 4.1 m over a scattering-vector range from 0.01 to 0.35 Å^−1^ [*q* = (4πsinθ)/λ, where 2θ is the scattering angle]. Data for protein samples at 1.5, 3.0, 6.1, 12.2, 24.3 and 48.6 mg ml^−1^ and buffer were measured at 283 K. No radiation damage was observed by comparison of 20 successive 0.05 s exposures. Data were processed and analyzed using the *ATSAS* package (Petoukhov *et al.*, 2012 ▶). SAXS data for the FnIII-3,4 MTSL-labelled mutants were measured and processed similarly using a detector distance of 3.1 m (0.01 < *q* < 0.44 Å^−1^). The ensemble-optimization method (EOM) was used to analyze interdomain flexibility (Bernadó *et al.*, 2007 ▶).

*Ab initio* shape reconstructions were calculated with *DAMMIF* (Franke & Svergun, 2009 ▶) and *DALAI_GA* (Chacón *et al.*, 2000 ▶). 20 independent models were generated with each program. Each set of structures were superimposed and an averaged model that represents the most populated volume within each set was calculated with *DAMAVER* (Volkov & Svergun, 2003 ▶). For representation, volumetric maps were calculated from the bead models with the *SITUS* package (Wriggers, 2010 ▶).

### 2.6. Site-directed spin labelling (SDSL)   

Cys substitutions, replacing Cys in the wild-type sequence or introducing new Cys residues, were created in the construct of the FnIII-3,4 fragment (1457–1666) by site-directed mutagenesis using the QuikChange method (Stratagene). The mutants were expressed at 288 K, were purified as for the wild-type protein and were labelled with the thiol-reactive paramagnetic nitroxide probe *S*-(1-oxyl-2,2,5,5-tetramethyl-2,5-dihydro-1*H*-pyrrol-3-yl)methyl methanesulfonothioate (MTSL). Proteins at 200 µ*M* in 20 m*M* sodium phosphate pH 7.5, 150 m*M* NaCl were incubated with a tenfold molar excess of MTSL for 1 h at room temperature and subsequently for 12 h at 277 K. Excess free MTSL was removed by extensive dialysis against the same buffer. Alternatively, for SAXS analysis of MTSL-labelled samples, excess free MTSL was removed and the proteins were equilibrated in 20 m*M* sodium phosphate pH 7.5, 150 m*M* NaCl, 5% glycerol by size-exclusion chromatography using a Superdex 200 10/300 GL column (GE Healthcare). The total free thiol groups before and after labelling were determined by titration with 5,5′-dithio-bis(2-nitrobenzoic acid) under denaturing conditions (García-Alvarez *et al.*, 2003 ▶) and revealed complete labelling.

### 2.7. Double electron–electron resonance (DEER) measurements and data analysis   

Solutions of MTSL-labelled proteins at a concentration between 100 and 200 µ*M* were mixed in a 1:1 volume ratio with deuterated glycerol, transferred into EPR quartz tubes and stored in liquid N_2_ until measurement.

Q-band DEER measurements were performed using a homemade spectrometer (Gromov *et al.*, 2001 ▶) working at a microwave frequency (mw) of ∼34.4 GHz and equipped with a 150 W travelling-wave tube (Applied Systems Engineering Inc). A homemade probe head that allows the measurement of up to 3 mm sample tubes (Tschaggelar *et al.*, 2009 ▶) was used. All experiments were performed at a temperature of 50 K using a continuous gas-flow cryostat refrigerated with He (Oxford Instruments).

The pulse sequence (π/2)mw_1_–τ_1_–(π)mw_1_–τ_1_–(π)mw_2_–τ_2_–(π)mw_1_–τ_2_–echo was set with mw pulse lengths of 12 ns for all pulses, and deuterium nuclear modulations were averaged out by adding eight experiments with τ_1_ increments of 16 ns. The pump mw frequency mw_1_ was set to the maximum absorption of the spectrum and the observer mw frequency mw_2_ was 80 MHz lower. The length of the experimental traces was between 3 and 6 µs depending on the transverse relaxation times. The time points were collected with a repetition rate of 300 Hz and the dipolar evolution traces were accumulated for several hours.

DEER traces were analyzed using *DeerAnalysis* (Jeschke *et al.*, 2006 ▶), which is available online at http://www.epr.ethz.ch/software/index. An exponential background decay function was fitted to the final part of the traces and was subtracted from the experimental trace. From the resulting dipolar time-evolution trace, the distance distribution was determined by Tikhonov regularization. A numerical value for the average distance between probes was then obtained for every pair of spin labels *i* (δ_*i*,EXP_)

Prediction of the populations of the conformations of the spin label attached to a certain position was performed from the structure of the individual domains using a library developed to model all possible conformations with a reduced number of significant rotamers (Polyhach *et al.*, 2011 ▶). This approach is implemented in the open-source package *MMM* (*Multiscale Modeling of Macromolecules*), a Matlab-based collection of routines accessible through a graphical user interface. Simulations of DEER experiments based on the protein structure and rotamer libraries were performed using the DEER window in *MMM*.

### 2.8. Molecular modelling   

To determine the relative arrangement of two solids, one needs to determine six relative coordinates: three for the translation degrees of freedom and three for the rotational degrees of freedom. To test the possible structural models against the experimental data, a complete set of relative arrangements was generated using a homemade Matlab-based program. The models were produced by combining a three-dimensional translational grid of 2 Å step size with a three-dimensional rotational grid containing pairs of angles α and β defining points uniformly distributed over a sphere (with every such orientation covering a solid angle of 0.0385 sr) plus an angle γ that was sampled every 10°. For every relative arrangement, the average position of the spin probe delivered by *MMM* was recalculated and the distances between average positions of the different spin pairs were computed (δ_*i*,MODEL_). In order to rank the models according to the degree of agreement with the experimental data, the parameter σ_DEER_, defined as follows, was calculated for every structural model,
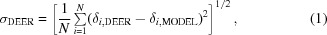
where *N* is equal to the number of interdomain distances that we have used for determination of the relative arrangements of the two FnIII domains.

Coordinates of the linker that connects FnIII-3 and FnIII-4 were constructed by a Monte Carlo approach that takes into account standard bond lengths and angles, a statistical distribution of backbone dihedral angles that conforms to residue-specific Ramachandran plots derived from the whole PDB (Hovmöller *et al.*, 2002 ▶) and the location by two DEER distance constraints of the spin label attached to C1559, a natural cysteine located in the central region of the linker. For every model of interdomain arrangement, backbone N, C^α^ and carbonyl C coordinates were computed from the backbone dihedrals using the Sugeta–Miyazawa algorithm (Sugeta & Miyazawa, 1967 ▶) and assuming standard peptide-bond lengths and angles. Carbonyl O atoms were added in the peptide plane. The initial rotation matrix was computed according to Shimanouchi & Mizushima (1955 ▶) from the backbone coordinates of the C-terminal residue of FnIII-3.

Linker models were rejected if they featured self-clashes or clashes with atoms in the FnIII-3 or FnIII-4 domains. Clashes were defined as an approach of two heavy atoms of nonconsecutive residues of closer than 2.5 Å.

Spin-label coordinates at residue C1559 were predicted by transforming the mean N—O midpoint coordinate in a rotamer library of an unrestricted MTSL side chain (Polyhach *et al.*, 2011 ▶) from the peptide standard frame to the local residue frame. A new σ_DEER_ parameter was calculated taking into account the interdomain distance constraints and also those obtained from measurements involving the linker cysteine C1559. The first 11 residues up to C1559 were modelled by the unrestricted Monte Carlo approach, whereas the remaining residues up to S1572 were modelled by a Monte Carlo Metropolis approach to steer the linker towards the N-terminal residue of domain FnIII-4, with the target coordinate being the C^α^ coordinate of this residue. Monte Carlo moves were accepted only if they moved the residue by at least 1.325 Å towards the target coordinate. This value led to an acceptable success rate in linker-model generation, with success being defined by an approach of the modelled S1572 C^α^ coordinate to the target coordinate of within 5 Å. The remaining difference was eliminated by evenly distributing it over all linker backbone atoms. Side groups were then added with *SCWRL*4 (Krivov *et al.*, 2009 ▶). The scattering curves of the atomic models were calculated and compared with the experimental SAXS profile with *CRYSOL* (Svergun *et al.*, 1995 ▶). Pair-distance distribution functions were calculated from atomic models with *HYDROPRO* (Ortega *et al.*, 2011 ▶).

### 2.9. Accession numbers   

The Protein Data Bank accession codes for the coordinates and structure factors of the FnIII-3 and FnIII-4 structures are 4wtw and 4wtx. SAXS data and models of wild-type FnIII-3,4 have been deposited in the Small Angle Scattering Biological Data Bank (SASBDB; Valentini *et al.*, 2014 ▶) under code SASDAT6.

## 3. Results   

### 3.1. Crystal structures of FnIII-3 and FnIII-4   

The individual structures of the FnIII-3 and FnIII-4 domains of β4 integrin were solved by X-ray crystallography. The structure of FnIII-3 (1457–1548) was refined against data to 1.60 Å resolution. The asymmetric unit contains two copies of FnIII-3 that are almost identical. After superimposition, the root-mean-square difference (r.m.s.d.) for all main-chain atoms between the two molecules is 0.59 Å. The structure of the FnIII-4 domain (1572–1666) was refined against data extending to 1.50 Å resolution; the FnIII-4 crystals contain a single protein molecule in the asymmetric unit. The FnIII-3 and FnIII-4 domains exhibit the canonical FnIII fold consisting of two β-sheets formed by strands ABE and C′CFG (strand G is divided into G1 and G2; Figs. 1 ▶ *b*–1 ▶ *e* and Supplementary Fig. S2)

A structure of FnIII-3 solved by NMR has been deposited in the PDB (PDB entry 2yrz; RIKEN Structural Genomics/Proteomics Initiative, unpublished work). Each of the 20 models of the NMR ensemble superposes well on the two FnIII-3 molecules of the crystal structure, with an r.m.s.d. ranging from 0.68 to 0.94 Å for all C^α^ atoms in the region 1457–1548. In spite of the overall similarity, there are significant differences between the solution and crystal structures (Supplementary Fig. S3). In our crystal structure β-strand A extends to A1468, similarly to as observed in other FnIII domains. In contrast, in the NMR structure the carbonyl group of S1467 is flipped and that of A1468 is rotated with respect to the crystal structure, impeding the formation of hydrogen bonds to β-strand B.

Superimposition of the crystal structures of the four FnIII domains of β4 reveals a conserved hydrophobic core that includes nine out of the 11 residues that are identical in the four domains. The polypeptide backbone is highly conserved in the β-strands and in loops A/B, E/F and F/G1 (Supplementary Fig. S4). On the other hand, the solvent-exposed surfaces of these domains show no similarities (for example, in their electrostatic properties; Supplementary Fig. S4*d*). In summary, the FnIII domains of β4 share a common scaffold, but each of them exhibits distinct external features that support functional specialization.

**Figure 2:**
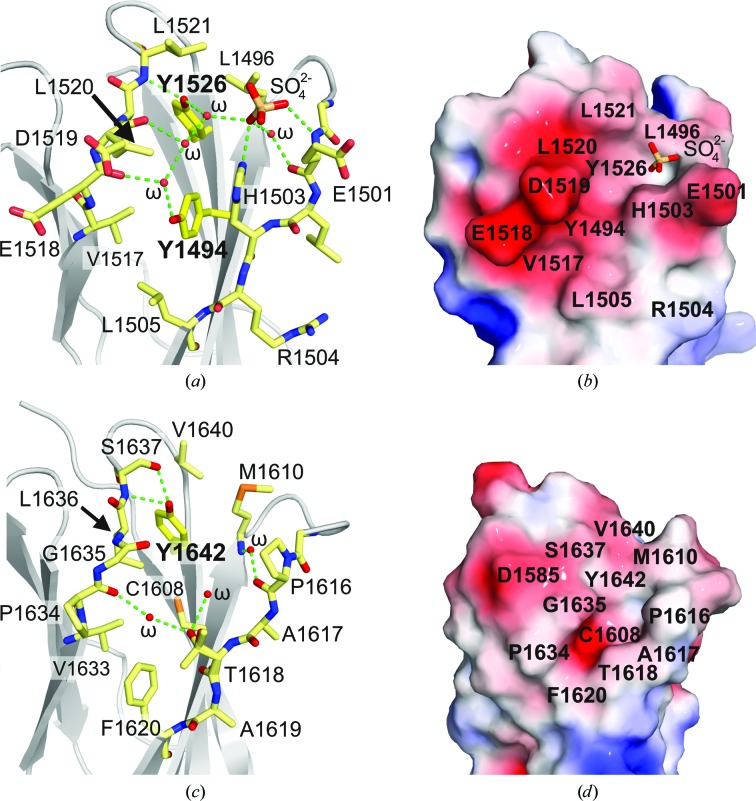
Structural environment of Tyr residues in FnIII-3 and FnIII-4. (*a*, *b*) Close-up of FnIII-3 around Y1494 and Y1526 in stick (*a*) and surface (*b*) representation coloured by the electrostatic potential on the surface from −3*kT*/e (red) to 3*kT*/e (blue). (*c*, *d*) Close-up of Y1642 in the FnIII-4 in stick (*c*) and surface (*d*) representation coloured by electrostatic potential as in (*b*).

Y1494 and Y1526 in FnIII-3, which are phosphorylated during signalling, are adjacent in the structure. Their aromatic rings are buried in the core of the domain, while the hydroxyl groups reach a cleft that contains a network of waters that is conserved in the two molecules of the asymmetric unit (Figs. 2 ▶ *a* and 2 ▶ *b*). Near Y1526, one of the FnIII-3 molecules has a sulfate ion coordinated by E1501, H1503 and two of the waters in the cleft. This anion is of interest because sulfates frequently mimic the phosphate group of phosphorylated residues. In the other FnIII-3 molecule in the asymmetric unit the position of the sulfate is occupied by the carboxylate of D1519 from a neighbouring molecule in the crystal. Collectively, the pocket near Y1526 has a propensity to bind negatively charged groups, despite being surrounded by the acidic residues E1501, E1518 and D1519. Finally, C1608 and Y1642 in FnIII-4 occupy positions equivalent to Y1494 and Y1526, respectively (Figs. 2 ▶ *c* and 2 ▶ *d*). In contrast to FnIII-3, the region around Y1642 is mostly uncharged.

### 3.2. Structure of the FnIII-3,4 region in solution   

**Figure 3:**
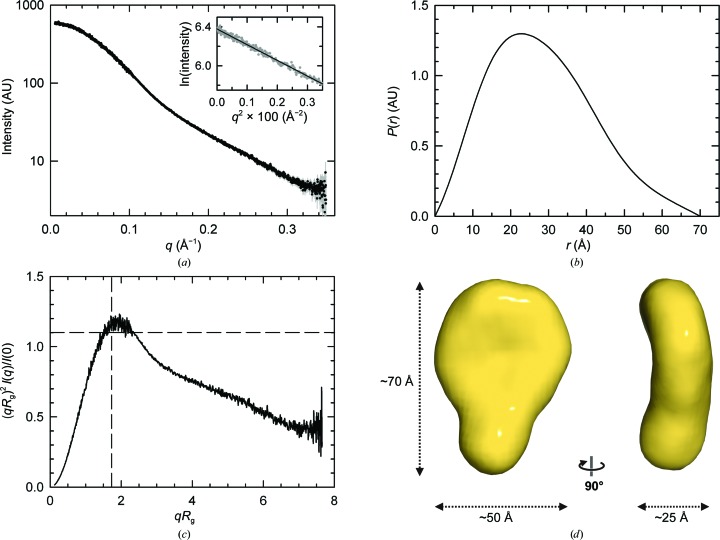
SAXS analysis of FnIII-3,4 of β4. (*a*) Scattering profile of FnIII-3,4 extrapolated to infinite dilution. The Guinier plot in the range 0.13 ≤ *qR*_g_ ≤ 1.30 is shown in the inset. (*b*) *P*(*r*) calculated from the SAXS data extending to 0.3 Å^−1^. (*c*) Dimensionless Krakty plot of the scattering data. The position of the maximum for compact globular particles is indicated by the cross-hair. (*d*) Orthogonal views of the *ab initio* molecular envelope obtained with *DAMMIN*; the structure is the average of 19 independent reconstructions.

Efforts to crystallize the complete FnIII-3,4 region of β4 were unsuccessful. Thus, we analyzed this fragment by SAXS, which provides information on the structure in solution at low resolution (Table 2 ▶). The scattering profile of FnIII-3,4 (residues 1457–1666) is shown in Fig. 3 ▶(*a*). The radius of gyration (*R*_g_) determined by Guinier analysis using data at very small scattering angles was 22.1 ± 0.8 Å. The same *R*_g_ value was obtained in the calculation of the pairwise distance distribution function, *P*(*r*), using data in the range of the scattering vector (*q*) from 0.01 to 0.30 Å^−1^. The *P*(*r*) of FnIII-3,4, which contains information about the size and shape of the particle, has a bell shape characteristic of globular particles and a maximum dimension (*D*_max_) of ∼70 Å (Fig. 3 ▶ *b*).

We used a dimensionless Kratky plot of the SAXS data to analyzed possible interdomain flexibility (Durand *et al.*, 2010 ▶). This representation of the FnIII-3,4 data has a maximum of ∼1.19 at *qR*_g_ ≃ 1.90, which is very close to the expected value for globular compact particles (maximum of ∼1.1 at *qR*_g_ ≃ 1.73), suggesting that this protein has very limited flexibility (Fig. 3 ▶ *c*).

Molecular envelopes of FnIII-3,4 were constructed from the SAXS data with *DAMMIF* and *DALAI_GA*, which implement two different *ab initio* algorithms. The normalized spatial discrepancy (NSD), a parameter that describes the agreement between three-dimensional models, was 0.67 ± 0.04 for 19 reconstructions generated with *DAMMIF* and 0.98 ± 0.03 for 20 models obtained with *DALAI_GA*. The NSD between the averaged models obtained with *DAMMIF* and *DALAI_GA* was 0.68. Thus, the two programs yield stable reconstructions of similar shapes. The molecular mass estimated from the excluded volume of the *DAMMIF* models (21.0 kDa) agrees with the mass calculated from the sequence (23.4 kDa), confirming that FnIII-3,4 is a monomer in solution. The *ab initio* envelopes resemble a slightly bended and elongated flat disc with overall dimensions of ∼70 × ∼50 × ∼25 Å (Fig. 3 ▶ *d* and Supplementary Fig. S5). In summary, analysis of FnIII-3,4 by SAXS revealed a compact structure in which the two FnIII domains might establish lateral inter­actions and have their longitudinal axes approximately lying on the same plane.

### 3.3. Determination of interdomain distances in the FnIII-3,4 region by SDSL and DEER using doubly spin-labelled samples   

**Figure 4:**
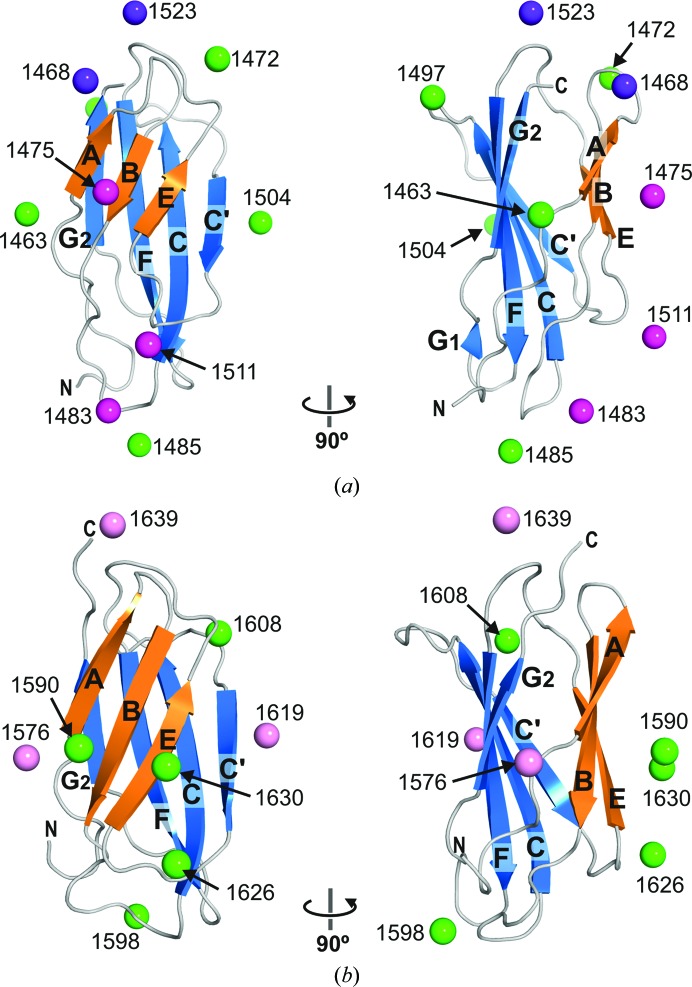
Paramagnetic labels attached to the FnIII-3 and FnIII-4 domains of β4. (*a*) The predicted average locations of the nitroxide group of MTSL attached to Cys are shown as spheres in the crystal structure of FnIII-3. The positions that yielded well defined inter-spin distances with a second probe at C1608 are coloured green. Those that resulted in broad distance distributions but did not alter the global structure of FnIII-3,4 are coloured magenta. The positions that resulted in a distortion of the structure are coloured dark violet. (*b*) Crystal structure of FnIII-4 with the estimated average position of the paramagnetic groups. The positions that yielded useful distances are coloured green, while those that resulted in broad distributions are shown in pink.

Owing to the globular shape of the individual domains, it was not possible to unambiguously dock the crystal structures of FnIII-3 and FnIII-4 into the SAXS-derived envelopes or to locate them by rigid-body fitting against the SAXS data. Therefore, to gather information on the relative orientation of the two FnIII domains we used EPR spectroscopy, since DEER experiments in combination with SDSL allow the determination of distances between selected residues in the range ∼10–80 Å (Jeschke, 2012 ▶), which fits the dimensions of the protein. We attached the MTSL paramagnetic probe to Cys either present in the integrin or engineered at solvent-exposed positions of FnIII-3 and FnIII-4 (Fig. 4 ▶ and Supplementary Figs. S6 and S7).

β4 contains three Cys residues in the FnIII-3,4 region: C1483 in FnIII-3, C1559 in the linker that connects the two FnIII domains and C1608 in FnIII-4. MTSL reacted with these three Cys residues when the wild-type protein was labelled.

**Figure 5:**
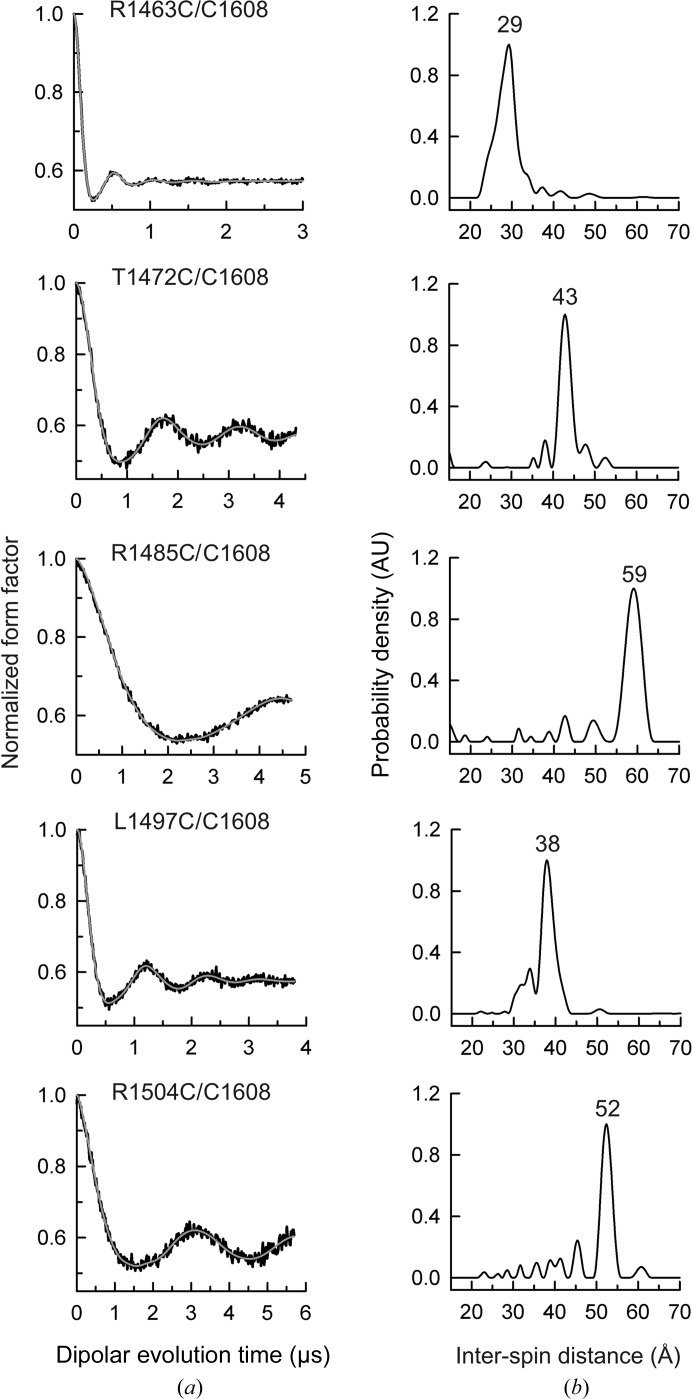
DEER results for the doubly labelled FnIII-3,4 mutants used to gather distances between C1608 in FnIII-4 and five positions in FnIII-3. (*a*) Normalized dipolar evolution (black lines) and fits to the data (grey lines) for each double Cys mutant. (*b*) Inter-spin distance distribution profiles calculated from the data in (*a*). The distances of the major peaks are indicated at the top.

To measure multiple interdomain distances between MTSLs attached to pairs of selected positions, each in one FnIII domain, we initially attempted to substitute the three wild-type Cys residues. The triple mutants C1483A/C1559A/C1608A and C1483S/C1559A/C1608S were insoluble when expressed in *E. coli*. On the other hand, the double mutant C1483S/C1559A was produced as a soluble protein and was used as a template to create nine triple β4 mutants that contain the wild-type C1608 and a second Cys engineered at a solvent-exposed position of FnIII-3. The purpose of this series of mutants was to locate C1608 with respect to FnIII-3. For clarity, we refer to the FnIII-3,4 mutants by the position of the Cys residue present. EPR analysis of the R1463C/C1608, T1472C/C1608, R1485C/C1608, L1497C/C1608 and R1504C/C1608 mutants yielded distance distributions that were characterized by single major narrow peaks centred at 29, 43, 59, 38 and 52 Å, respectively (Fig. 5 ▶, Table 3 ▶). This indicates that the spin label at residue 1608 occupies a relatively fixed position with respect to FnIII-3, suggesting low conformational variability of the label (as predicted by modelling; see Supplementary Fig. S7) and very limited interdomain wiggling. SAXS analysis of these mutants labelled with MTSL revealed that they have the same overall structural parameters as the wild-type protein (Supplementary Figs. S8*a* and S8*b*), confirming that the mutations and subsequent labelling did not distort the global structure of the fragment.

Two other mutants in this series, R1475C/C1608 and A1511C/C1608, showed SAXS-derived *R*_g_, *D*_max_ and *P*(*r*) values similar to those of wild-type FnIII-3,4, yet DEER analysis revealed broad inter-spin distance distributions (Supplementary Figs. S8*c*–S8*f*) that were not used for structural modelling of the fragment. Finally, SAXS analysis of two additional mutants, A1468C/C1608 and N1523C/C1608, showed significantly larger *R*_g_ (26.1 and 26.1 Å) and *D*_max_ (∼88 and ∼92 Å) values than the wild type and an apparently large flexibility (Supplementary Figs. S8*c*–S8*g*), which suggests a distorted interdomain arrangement. Furthermore, DEER analysis of these mutants showed multiple inter-spin distance peaks and consequently these distances were not used for rigid-body fitting. In summary, Ala1468 and Asn1523, which are ∼14 Å apart on the FnIII-3 surface, define an area that is important for maintaining the organization of the FnIII-3,4 structure.

Next, we attempted to obtain the position of C1483 in FnIII-3 with respect to FnIII-4 following the same approach. For this, we created mutants with the C1608A substitution, which had C1483 and an engineered Cys at solvent-exposed positions of FnIII-4 (Supplementary Table S1). However, DEER analysis of the mutants yielded broad and multimodal distance distributions (Supplementary Fig. S9). Modelling of MTSL attached to C1483 with *MMM* indeed confirms that labelling of this residue is unfavourable and could distort the structure. Nevertheless, replacing the label at the C1483 position by a label at R1485C did not solve the problem because the protein mutants were either insoluble or yielded very wide inter-spin distance distributions (Supplementary Fig. S9).

### 3.4. Determination of additional interdomain distances by DEER using triple spin-labelled FnIII-3,4 mutants   

In case C1608 was important to preserve the native-like structure, we decided to maintain this residue and designed mutants that contain three Cys residues and carry C1483S/C1559A substitutions. These proteins include a Cys pair of C1608 in FnIII-4 and a Cys in FnIII-3 which had yielded well defined inter-spin distances in the double-labelled mutants. In addition, they contain a new Cys engineered at solvent-exposed positions of FnIII-4. We generated the eight triple Cys mutants collected in Table 3 ▶. All of these proteins were produced in a soluble form; they were labelled with MTSL and analyzed by SAXS and DEER. Analysis of the SAXS profiles of these mutants labelled with MTSL revealed that they are monomeric in solution and that they have an overall structure compatible with that of the wild-type protein (Supplementary Figs. S10*a* and S10*b*).

**Figure 6:**
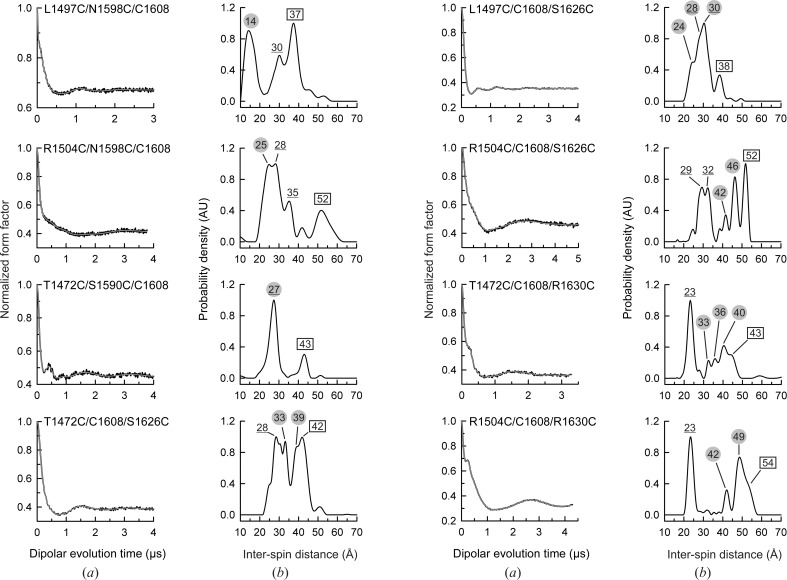
DEER results for triply labelled FnIII-3,4 mutants. (*a*) Normalized dipolar evolution (black lines) and fits to the data (grey lines) for each of eight triply labelled mutants. (*b*) Distance distribution profiles calculated from the data in (*a*). The positions of peaks that correspond to distances previously observed in doubly labelled mutants containing C1608 are shown in rectangles. The positions of peaks assigned to intradomain distances modelled on the FnIII-4 structure are underlined. Finally, the peaks attributed to interdomain distances that do not involve C1608 are shown in grey circles. See Supplementary Fig. S10 for analysis of these proteins by SAXS.

Analysis of the triply labelled mutants by DEER is expected to yield information on three inter-spin distances. Firstly, an intradomain distance between the probes at C1608 and at a second Cys in FnIII-4; secondly, an interdomain distance between the probes attached to each of the engineered Cys residues in FnIII-3 and C1608 in FnIII-4; and finally, a second interdomain distance between the probes attached at the Cys engineered in both domains, which is the distance that we want to use to determine the orientation of the domains. In addition to the three inter-spin distances, DEER measurements of objects containing three spin labels have been reported to show artifacts or ghost distances (von Hagens *et al.*, 2013 ▶). In order to avoid and identify the presence of such artifacts, the measurements were performed at lower inversion efficiencies for the pump pulse of mw_2_ (pump pulse of turning angle π, π/2 and π/4). No ghost peaks could be identified for any of the samples; for this reason, the spectra shown in Fig. 6 ▶ are those obtained with the normal DEER sequence containing a π pump pulse.

In general, the DEER spectra of the triply labelled mutants showed several peaks that were broader than in the doubly labelled mutants. In order to identify the interdomain distance of interest, the peaks corresponding to the other distances were assigned. The FnIII-4 intradomain distances (underlined values in Fig. 6 ▶) were estimated by modelling the conformation of the probes with *MMM* using the crystal structure of FnIII-4. The inter-domain distances involving C1608 (values in square boxes in Fig. 6 ▶) could be identified from the measurements of double-labelled mutants. By exclusion, the remaining peaks could be assigned to new interdomain distance distributions (values surrounded by circles in Fig. 6 ▶). Whenever two or more peaks were assigned to the interdomain distance not involving C1608, an average value for the distance was taken for the structure modelling.

Peaks in the DEER inter-spin distance distributions of the eight triply labelled mutants were assigned to pairs of labelling positions as follows. The inter-spin distance distribution of the L1497C/N1598C/C1608 mutant shows three peaks at ∼14, ∼30 and ∼37 Å. Modelling of MTSL at 1598–1608 within FnIII-4 predicts a bimodal inter-spin distance at 27 and 34 Å, which is likely to correspond to the observed peak centred at 30 Å. The experimentally observed distance between the 1497–1608 pair was 37 Å. Hence, the distance peak at ∼14 Å was assigned to the 1497–1598 pair.

The distance distribution obtained for the R1504C/N1598C/C1608 mutant shows peaks at 25, 28, 35 and 52 Å. As described above, the peaks at 28 and 35 Å were assigned to the intradomain pair 1598–1608. The peak at 52 Å corresponds to the 1504–1608 pair. Thus, the peak at 25 Å was assigned to the 1504–1598 pair.

The T1472C/S1590C/C1608 mutant yielded a distance distribution with two peaks at 27 and 43 Å. The first peak corresponds to the estimated distance between the probes at residues 1590 and 1608, while the spacing at 43 Å had previously been observed for the 1472–1608 pair. Owing to the large integrated intensity of the peak at 27 Å, this distance was also assigned to the 1472–1590 pair.

The distance distribution of the T1472C/C1608/S1626C mutant has peaks at 28, 33, 39 and 42 Å. The distance between the intradomain pair 1608–1626 was estimated to be 28 Å. The 42 Å peak corresponds to the 1472–1608 pair. Therefore, the doublet at 33 and 39 Å was assigned to the 1472–1626 pair.

The distance distribution of the L1497C/C1608/S1626C mutant has peaks at 24, 30 and 38 Å and a shoulder at ∼28 Å. The estimated distance between the 1608–1626 pair (28 Å) is likely to correspond to the peaks at 28–30 Å. The distance between the 1497–1608 pair was already observed at 38 Å in the doubly labelled mutant (L1497C/C1608). Finally, the pair 1497–1626 was assigned to the triplet at 24, 28 and 30 Å.

The distance distribution of the R1504C/C1608/S1626C mutant has peaks at 29 and 32 Å that are similar to the estimated distance between the probes modelled at residues 1608 and 1626 in FnIII-4. There is also a peak at 52 Å that corresponds to the distance measured for the 1504–1608 pair. Finally, two peaks at 42 and 46 Å were attributed to the 1504–1626 pair.

The T1472C/C1608/R1630C mutant produced a distance distribution with a major peak at 23 Å and several peaks in the range 33–43 Å. The distance at 23 Å matches the estimated separation of the nitroxide groups of the pair 1608–1630. On the other hand, the 1472–1608 pair yields a distance peak at 43 Å in previous doubly and triply labelled mutants. Hence, the triplet at 33, 36 and 40 Å was assigned to the distance of the 1472–1630 pair.

Finally, the distance distribution obtained for the R1504C/C1608/R1630C mutant shows the peak at 23 Å attributed to the 1608–1630 pair and peaks at 42 and 49 Å. The distance of the 1504–1608 pair, expected to be 52 Å, appears as a shoulder at 54 Å. Therefore, the doublet at 42 and 49 Å was assigned to the 1504–1630 pair.

In summary, analysis of the triply MTSL-labelled FnIII-3,4 proteins yielded eight nonredundant interdomain distances between the spin probe attached to pairs of residues different from C1608. All distances and their assignments are collected in Table 3 ▶.

### 3.5. DEER measurement of distances between C1559 in the interdomain linker and the FnIII domains   

**Figure 7:**
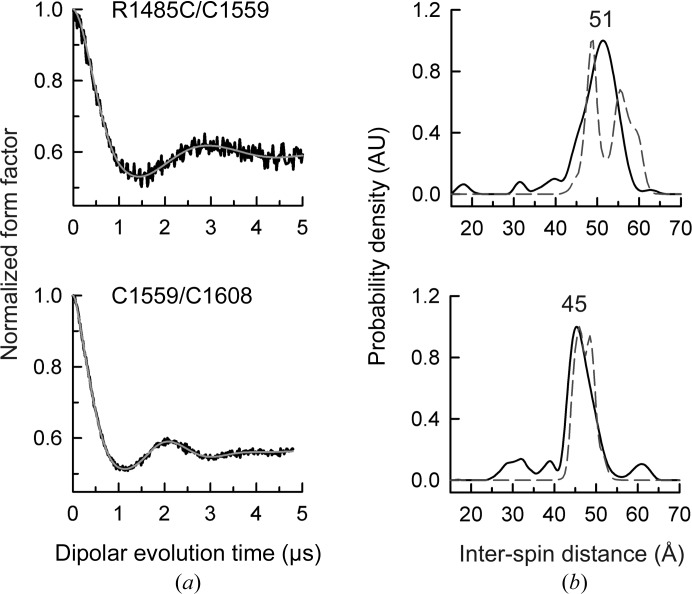
DEER results for mutants containing C1559 in the interdomain linker. (*a*) Normalized dipolar evolution (black lines) and fits to the data (grey lines) for two doubly labelled mutants that include C1559. (*b*) Distance distribution profiles calculated from the data in (*a*) (solid line) and a calculated distribution for one of the final models (dashed line). See Supplementary Fig. S10 for analysis of these proteins by SAXS.

To obtain information on the position of the linker that connects the two FnIII domains, we measured distances between MTSL probes attached to C1559, located at the centre of the linker, and to a second Cys in either FnIII-3 or FnIII-4. We used the single mutant C1483A that contains the Cys pair C1559/C1608 present in the wild-type sequence and the triple mutant C1483A/R1485C/C1608A that contains the pair R1485C/C1559. The structural parameters estimated from the SAXS profiles of these two mutants labelled with MTSL revealed that they have similar *R*_g_, *D*_max_ and *P*(*r*) values to the wild-type protein (Supplementary Figs. S10*c* and S10*d*). The distance distributions derived from the DEER data of these two mutants are characterized by single major peaks centred at 51 Å (R1485C/C1559) and 45 Å (C1559/C1608) (Fig. 7 ▶ and Table 3 ▶) that can be explained by considering a fixed anchor for the probe at C1559, indicating that the linker might have very moderate flexibility.

### 3.6. Modelling the structure of the FnIII-3,4 region by combining crystal structures, SAXS and DEER-derived distance constraints   

13 interdomain distances derived from the DEER measurements (Table 3 ▶) were used to derive the relative position of the FnIII-3 and FnIII-4 domains (Supplementary Fig. S11). Each distance value in the table corresponds to the average distance between the paramagnetic groups in all possible conformations of the MTSLs at the two labelled positions. Owing to the large conformational flexibility of the probe, for a single labelling site the paramagnetic groups could occupy positions more than 1 nm apart from each other. To reduce the uncertainty in the location of the N—O group, the conformation distribution of MTSL was computed for every labelled position using a rotamer-library approach and the crystal structures of the individual domains (Supplementary Fig. S6 and S7); the average coordinates of the paramagnetic centre were then calculated.

**Figure 8:**
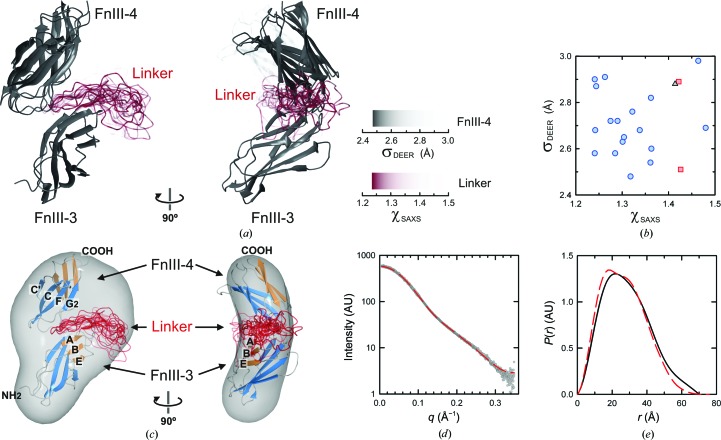
Structure of the FnIII-3,4 region derived from a combination of crystal structures, DEER, SAXS and modelling methods. (*a*) Two views of the ensemble of models of FnIII-3,4 that fit the DEER and SAXS data. The orientations of FnIII-4 are coloured according to the fit to the 15 DEER restraints (σ_DEER_) of the best models once the linker had been built. The colour intensity of the models of the linker (red) denotes the fit to the SAXS data (χ_SAXS_) of the FnIII-3,4 structures. The σ_DEER_ and χ_SAXS_ colour scales are shown. (*b*) Plot of the σ_DEER_ and χ_SAXS_ values determined for the 21 models that best fit to the experimental data. χ_SAXS_ was determined for *q* ≤ 0.3 Å^−1^. Points with the same symbol correspond to models with the same orientation of FnIII-4. The SAXS profile is sensitive to the conformation of the linker, as revealed by the wide spread of χ_SAXS_ values for a given arrangement of the two FnIII domains. (*c*) Docking of the atomic models to the SAXS-derived averaged molecular envelope. For clarity, only the most frequent FnIII-4 orientation and the corresponding modelled linkers are shown. (*d*) Comparison of the theoretical scattering curve of the model with lowest χ_SAXS_ (red dashed line) to the experimental SAXS data (grey dots). (*e*) *P*(*r*) functions calculated for the atomic model (red dashed line) and estimated from the SAXS data (black line). Details of the modelling of the inter-domain orientation are shown in Supplementary Fig. S11.

To explore the space of relative arrangement between the two FnIII domains, FnIII-3 was fixed and the position of FnIII-4 was swept using an exhaustive six-dimensional search. Only orientations with a discrepancy between the calculated and experimental DEER distances (parameter σ_DEER_) of ≤3 Å were accepted. This produced a set of relative arrangements compatible with the DEER restraints. These structures were grouped into 24 clusters defined by a maximum r.m.s.d. of 4 Å for the position of all of the backbone atoms within each cluster, from which one representative structure was selected. Then, for each representative structure, multiple conformations of the 23-residue linker that connects the FnIII-3 and FnIII-4 domains were built using native-like geometry. For every resulting model of the FnIII-3,4 region, the SAXS profile and the parameter σ_DEER_ were calculated, including the two DEER-distance restraints involving C1559. The plausible models were selected if the parameter χ_SAXS_, the discrepancy between the calculated and the experimental SAXS curves, was below 1.5 and σ_DEER_ was ≤3 Å (Figs. 8 ▶ *a* and 8 ▶ *b*). It turned out that of 21 selected models for the structure of the complete fragment, 18 of them correspond to the same relative arrangement of FnIII-3 and FnIII-4, differing only in the structure of the linker. The resulting composite atomic structures fit into the reconstructions created *ab initio* from the SAXS data, the scattering curves calculated for the models match the SAXS profile and the calculated *P*(*r*) of the models reproduce the distribution estimated from the experimental scattering curve (Figs. 8 ▶ *c*, 8 ▶ *d* and 8 ▶ *e*).

### 3.7. Structure of FnIII-3,4 of integrin β4   

The relative orientations of the FnIII-3 and FnIII-4 domains were similar in the refined models. The longitudinal axes of the two domains form an angle of ∼170°, resulting in a slightly bent structure. The ABE β-sheet of FnIII-3 and the C′CFG β-sheet of FnIII-4 are oriented towards the concave or inner side of the structure. Strands A and G2 at one edge of the β-sandwich of FnIII-3 face a region of FnIII-4 formed by the B/C and F/G2 loops and the N-terminus.

The linker occupies a region near the A/B and E/F loops at one tip of FnIII-3. Notably, A1468 and N1523, substitution of which by Cys-MTSL distorted the FnIII-3,4 structure (see above), are located in strand A and loop E/F of FnIII-3, respectively, near the estimated position of the linker. Thus, mutation of A1468 and N1523 is likely to distort the structure by altering primarily the organization of the linker. The modelled position of the linker and the small contact between the two domains support the linker being required for the correct arrangement of the FnIII-3 and FnIII-4 domains.

**Figure 9:**
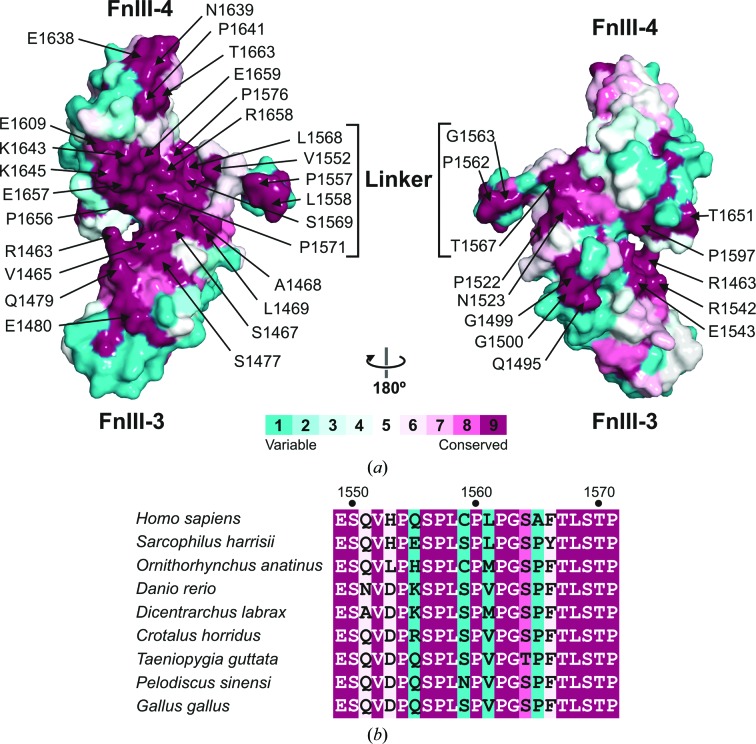
Conserved interdomain region in FnIII-3,4 of β4. (*a*) Molecular surface of the FnIII-3,4 structure coloured by the evolutionary conservation of the amino acids in the β4 sequences of 39 species calculated with the *ConSurf* server. The linker is shown in the conformation that yielded the best fit to the SAXS data. (*b*) Alignment of the sequences of the linker of nine species representative of the evolutionary variability. Positions are coloured according to the evolutionary conservation as in (*a*). A complete alignment of the sequences of the β4 FnIII-3,4 region of 39 species is shown in Supplementary Fig. S12.

FnIII-3,4 mediates interactions with several proteins, yet no specific binding sites have been mapped in this region. The frequent conservation of functional sites in proteins prompted us to investigate whether the FnIII-3,4 structure contains evolutionary conserved patches that could correspond to potential interaction sites. Analysis of the sequences of integrin β4 from mammals, birds, reptiles and fishes revealed an evolutionarily conserved surface spanning FnIII-3 and FnIII-4 at the inner side of the structure (Fig. 9 ▶). In addition, the final residues of the linker (^1567^TLSTP^1571^), which are also fully conserved, are predicted to lie between the two domains at the inner side. On the other hand, the outer surface is poorly conserved. In summary, the two FnIII domains and the linker form a continuous conserved surface that might correspond to an area of functional relevance.

## 4. Discussion   

Large proteins with relatively long flexible linkers between rigid domains are often hard to crystallize. Solution structures of such proteins can be characterized by combining atomic resolution information on the local structure of the domains with lower-resolution information on global structure. Such approaches have been demonstrated by combining X-ray structures of the domains with DEER, SAXS and FRET data for the ESCRT-I (Boura *et al.*, 2011 ▶) and ESCRT-II (Boura *et al.*, 2012 ▶) complex and by combining NMR and DEER data to solve the interdomain arrangement in the Omp85 protein BamA (Ward *et al.*, 2009 ▶) and the structure of the RsmE–RsmZ protein–RNA complex (Duss, Michel *et al.*, 2014 ▶; Duss, Yulikov *et al.*, 2014 ▶). By providing distance distributions rather than only mean distances, the DEER data allow the recognition of large-scale distributions of conformations, as is the case for ESCRT-I, ESCRT-II and RsmE–RsmZ, or to conclude that the relative domain orientation is well defined, as is the case for BamA and FnIII-3,4 of β4. Although the X-ray structures of the individual domains and the DEER data on their own are sufficient to generate a model ensemble, we find that this ensemble is further constrained by the SAXS data with respect to both relative domain orientations and linker conformations. The approach introduced here allows quantitative estimation of the uncertainty of the structural model and is extensible to systems with multiple domains. Each added domain requires of the order of 12 additional DEER distribution constraints. The number of constraints required to specify a linker conformation depends on the linker length. At least two constraints per reference point between ten-residue segments of the linker are required to obtain a coarse ensemble. We expect that localization approaches, such as that demonstrated for lipoxygenase (Gaffney *et al.*, 2012 ▶), will allow narrowing of the ensemble. Recent work on the pro-apoptotic protein Bax indicates that five DEER constraints per reference point provided good localization (Bleicken *et al.*, 2014 ▶).

The compact structure of FnIII-3,4 revealed here has implications for understanding the organization and inter­actions of integrin β4. The region of β4 formed by the CS, FnIII-3,4 and the C-tail is believed to adopt a folded-back structure. Intramolecular FRET between cyan (CFP) and yellow (Venus) fluorescent proteins has been detected in cells expressing a β4 construct that incorporates Venus right upstream of FnIII-3 and the CFP at the C-terminus (Frijns *et al.*, 2012 ▶). In our structure, the N- and C-termini of FnIII-3,4 are ∼60–65 Å apart, which is within the distance range that allows FRET between the CFP–Venus pair (<100 Å). In addition, since the CFP was at the end of the β4 chain in this FRET sensor, the 86-residue C-tail might be projected towards the CS, contributing to the proximity of the CFP and Venus tags. We observed negligible interdomain conformational variability, which suggests that FnIII-3,4 does not behave as a flexible hinge that could bend in response to CS/C-­tail interactions. On the contrary, FnIII-3,4 probably acts as a rigid platform that promotes proximity of the CS and the C-tail.

FnIII-3,4 and the final portion of the CS (region 1436–1667) are sufficient for binding to BPAG1e (Koster *et al.*, 2003 ▶). The last 21 residues of the CS do not alter the structure of FnIII-3,4, as observed by SAXS analysis of the 1436–1666 fragment (Manso & de Pereda, unpublished results). Therefore, our structure is in a BPAG1e binding-active conformation of FnIII-3,4, which is likely to be the organization of this region in the complete α6β4 integrin. Interaction of β4 with BPAG1e requires both FnIII-3 and FnIII-4, suggesting that the binding interface extends along the two domains. It is notable that the evolutionarily conserved surface in FnIII-3,4, which probably corresponds to a functional site, spreads throughout the two FnIII domains and the linker and could be involved in binding to BPAG1e. In fact, the clustering of conserved residues in FnIII-3,4 is reminiscent of the plectin-binding site in β4, which is the largest surface of FnIII-1,2 that is preserved among multiple species (Supplementary Fig. S13).

Three Tyr residues in FnIII-3,4 (Y1494, Y1526 and Y1642) have been implicated in phosphorylation-dependent signalling. Phospho-Y1494 is recognized by the SH2 domain of SHP2 (Bertotti *et al.*, 2006 ▶), while the phosphotyrosine-binding (PTB) domain of Shc binds to phosphorylated Y1526 and possibly Y1642, which are within N*XX*pY motifs (where *X* is any amino acid; Dans *et al.*, 2001 ▶). Phospho-Tyr ligands adopt extended conformations when bound to SH2 domains; similarly, residues upstream of N*XX*pY motifs make β-sheet contacts with PTB domains (Kaneko *et al.*, 2012 ▶). Therefore, the structural environments of Y1494, Y1526 and Y1642 are not compatible with binding to SH2 or PTB domains. This apparent contradiction can be reconciled if the FnIII fold is disrupted in the Tyr-phosphorylated form of β4. For example, mechanical stretching of β4 could expose cryptic Tyr-phosphorylation sites. Alternatively, phosphorylation of Tyr could trigger unfolding of the FnIII domain or could prevent its refolding. The sulfate near Y1526 in the crystal structure suggests that FnIII-3 might tolerate phosphorylation at this position. Nonetheless, owing to the small exposure of Y1494 and Y1526 to the solvent, phosphates attached to these residues are likely to cause steric hindrance. The nearby acidic residues E1518, D1519 and E1501 could also create electrostatic repulsion with the phosphates. In addition, owing to the proximity between Y1494 and Y1526, simultaneous phosphorylation of these Tyr residues could destabilize FnIII-3 owing to repulsion between the phosphate groups, suggesting that multiple phosphorylation events might collaborate to fully trigger β4-dependent signalling. It is also possible that phosphorylation of one residue might act as an initiator, relaxing the structure and favouring the phosphorylation of the companion Tyr in a sequential manner. Interestingly, Y1494 has been proposed as a main regulator of α6β4 signalling in cancer cells (Dutta & Shaw, 2008 ▶). In contrast to Y1494 and Y1526 in FnIII-3, Y1642 in FnIII-4 sits in a wider pocket surrounded by uncharged residues. Therefore, phosphorylation of Y1642 is less likely to destabilize FnIII-4 and correlates with a minor role of Y1642 in the recruitment of Shc (Dans *et al.*, 2001 ▶).

In summary, FnIII-3,4 emerges as a structural and functional unit within the β4 integrin. Global changes in the interdomain arrangement, for example in response to pulling forces, could alter or unveil binding sites in FnIII-3,4, suggesting that α6β4 might act as a mechanosensor. Conversely, local changes in one of these FnIII domains could propagate to other regions of β4, such as the CS and C-tail. This work provides a detailed structural framework to better investigate the role β4 in epithelial homeostasis and in carcinoma progression.

## 5. Related literature   

The following references are cited in the Supporting Information for this article: de Pereda, Lillo *et al.* (2009 ▶), Robert & Gouet (2014 ▶) and Sievers *et al.* (2011 ▶).

## Supplementary Material

PDB reference: third FnIII domain of integrin β4, 4wtw

PDB reference: fourth FnIII domain of integrin β4, 4wtx

Supporting Information.. DOI: 10.1107/S1399004715002485/kw5120sup1.pdf

## Figures and Tables

**Table 1:** Summary of crystallographic analysis Values in parentheses are for the outer resolution shell.

Protein	FnIII-3 (14571548)	FnIII-4 (15721666)		
Data set	Native	Native 1	EMTS soak	Native 2
Data collection				
Space group	*I*2_1_2_1_2_1_	*P*4_1_2_1_2	*P*4_1_2_1_2	*P*4_1_2_1_2
Unit-cell parameters ()				
*a*	66.7	56.9	56.9	57.3
*b*	68.3	56.9	56.9	57.3
*c*	88.4	78.4	78.4	76.3
Wavelength ()	1.5418	1.5418	1.5418	0.9793
Resolution ()	1.60 (1.691.60)	1.80 (1.901.80)	1.95 (2.051.95)	1.50 (1.541.50)
Unique reflections	26721	11858	17812†	19975
Average multiplicity	9.0 (8.1)	19.7 (20.1)	15.0 (14.6)	24.3 (26.3)
Completeness (%)	98.6 (91.2)	95.0 (89.5)	99.6 (99.9)	95.3 (99.8)
*R*_meas_ ‡	0.039 (0.313)	0.052 (0.609)	0.072 (0.420)	0.062 (1.433)
CC_1/2_	1.00 (0.96)	1.00 (0.98)	1.00 (0.98)	1.00 (0.89)
*I*/(*I*)	41.7 (7.7)	45.1 (7.8)	32.4 (8.8)	33.9 (3.3)
SIRAS phasing				
*R*_iso_ §			0.344	
Phasing power (iso, acentric/iso, centric/ano)			2.38/2.06/2.36	
FOM (acentric/centric)			0.59/0.51	
Model refinement				
No. of protein molecules in asymmetric unit	2			1
Resolution range ()	351.60			461.50
Unique reflections (work/free)	25369/1340			18911/992
*R*_work_/*R*_free_ ¶	0.151/0.180			0.195/0.218
No. of non-H atoms				
Protein††	826/822			773
Waters	222			122
Sulfates	10			
PEGs	20			
Average *B* values (^2^)				
Wilson plot	14.2			20.0
Protein††	19.2/20.9			28.5
Waters/sulfates/PEGs	30.2			34.8
Sulfates	31.3			
PEGs	27.6			
R.m.s.d. from ideal geometry‡‡				
Bond lengths ()	0.009			0.012
Angles ()	1.2			1.3
R.m.s.d. *B* factors§§ (^2^)				
Main chain	1.5			3.9
Side chain	3.4			6.2
Ramachandran plot¶¶				
Residues in favoured regions	234 [98.3]			108 [98.2%]
Additionally allowed	4 [1.7%]			2 [1.8%]
Outliers	0 [0.0%]			0 [0.0%]
Side-chain rotamer outliers¶¶	1 [0.5%]			1 [1.2%]
Clashscore¶¶	0.60			0.66
PDB code	4wtw			4wtx

† Keeping Bijvoet pairs separate.

‡ The redundancy-independent *R* factor *R*_meas_ = 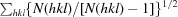 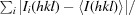 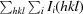 , where *I_i_*(*hkl*) is the *i*th measurement and *I*(*hkl*) is the mean of all measurements of *I*(*hkl*) (Diederichs Karplus, 1997 ▶).

§ *R*_iso_ = 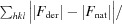 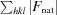, where *F*_der_ is the heavy-atom-derivative structure factor and *F*_nat_ is the protein structure factor.

¶ *R*_work_ = 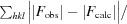 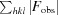; *R*_free_ was calculated using a randomly chosen 5% of reflections that were not included in the refinement and *R*_work_ was calculated for the remaining reflections.

†† Values for each protein chain.

‡‡ Referred to the ideal geometry defined by Engh Huber (1991 ▶).

§§ Calculated for bonded atom pairs with *MOLEMAN*2 (Kleywegt, 1997 ▶).

¶¶ Values were obtained using *MolProbity* (Chen *et al.*, 2010 ▶).

**Table 2:** SAXS data collection and derived parameters for 4 FnIII-3,4

Data-collection parameters	
Instrument	EMBL P12 beamline, PETRA, DESY, Hamburg
Beam geometry	0.2 0.12mm
Wavelength ()	1.24
*q* range (^1^)	0.010.35
Exposure time (s)	20 0.05
Concentration range (mgml^1^)	1.548.6
Temperature (K)	283
Structural parameters†	
*I*(0)/*c* [Table-fn tfn10] (10^2^cm^2^mg^1^) (from Guinier)	1.24 0.01
*R*_g_ () (from Guinier)	22.1 0.8
*I*(0)[Table-fn tfn10] (10^2^cm^2^mg^1^) [from *P*(*r*)]	1.23 0.01
*R*_g_ () [from *P*(*r*)]	22.0
*D*_max_ ()	70
Porod volume estimate *V*_p_ (^3^)	32000 1000
Excluded volume estimate *V*_ex_ (^3^)	42000 200
Molecular-mass determination	
*I*(0)[Table-fn tfn10] (10^2^cm^2^mg^1^)	4.20 0.01
Molecular mass *M*_r_ [Table-fn tfn11] (Da) [from *I*(0)]	19200
Molecular mass *M*_r_ [Table-fn tfn11] (Da) [from (*V*_p_/1.6)]	20000 625
Molecular mass *M*_r_ [Table-fn tfn11] (Da) [from (*V*_ex_/2)]	21000 100
Calculated monomeric *M*_r_ from sequence	23360
Software used	
Primary data reduction	Automated SAXS pipeline
Data processing	*PRIMUS* (Konarev *et al.*, 2003 ▶)
*Ab initio* analysis	*DAMMIF* (Franke Svergun, 2009 ▶), *DALAI_GA* (Chacn *et al.*, 2000 ▶)
Validation and averaging	*SUPCOMB* (Kozin Svergun, 2001 ▶) *DAMAVER* (Volkov Svergun, 2003 ▶)
Computation of model intensities	*CRYSOL* (Svergun *et al.*, 1995 ▶)
Three-dimensional graphical representations	*PyMOL* (Schrdinger)
SASBDB code	SASDAT6

† Values reported for the data extrapolated to infinite dilution.

‡ Absolute intensities determined using water as a secondary standard (Orthaber *et al.*, 2000 ▶).

§ Calculated using BSA as a standard (66000Da).

**Table 3:** Inter-spin distances and structural parameters of FnIII-3,4 mutants labelled with MTSL

	Spin-labelled sites				Structural parameters†	
FnIII-3,4 mutant	FnIII-3	Linker	FnIII-4	Average inter-spin distance ()	*R*_g_ ()	*D*_max_ ()
R1463C/C1608‡	1463		1608	29	22.5	70
T1472C/C1608‡	1472		1608	43	22.6	70
R1485C/C1608‡	1485		1608	59	22.2	70
L1497C/C1608‡	1497		1608	38	22.7	70
R1504C/C1608‡	1504		1608	52	22.3	70
L1497C/N1598C/C1608‡			1598, 1608	30 (27/34)§	22.6	70
	1497		1598	14		
	1497		1608	37		
R1504C/N1598C/C1608‡			1598, 1608	28/35 (27/34)§	22.3	70
	1504		1608	52		
	1504		1598	25		
T1472C/S1590C/C1608‡			1590, 1608	27 (25)§	22.7	70
	1472		1608	43		
	1472		1590	27		
T1472C/C1608/S1626C‡			1626, 1608	28 (28)§	22.8	70
	1472		1608	42		
	1472		1626	33/39¶		
L1497C/C1608/S1626C‡			1626, 1608	28/30 (28)§	22.8	70
	1497		1608	38		
	1497		1626	24/28/30¶		
R1504C/C1608/S1626C‡			1626, 1608	29/32 (28)§	22.7	70
	1504		1608	52		
	1504		1626	42/46¶		
T1472C/C1608/R1630C‡			1630, 1608	23 (23)§	22.7	70
	1472		1608	43		
	1472		1630	33/36/40¶		
R1504C/C1608/R1630C‡			1630, 1608	23 (23)§	22.6	70
	1504		1608	54		
	1504		1630	42/49¶		
R1485C/C1559††	1485	1559		51	22.3	70
C1559/C1608‡‡		1559	1608	45	21.7	70

† Determined from the SAXS profile by indirect transform using data for *q* 0.3^1^.

‡ Contains the substitutions C1483S and C1559A.

§ Numbers in parentheses indicate the position of the maximum of the distance distribution estimated by modelling the MTSL probe on the crystal structure of the FnIII-4 domain.

¶ For distances with multi-modal distributions, which are likely to correspond to alternative conformations of the probe, the mean value was used for rigid-body fitting.

†† Contains the substitutions C1483A and C1559A.

‡‡ Contains the single mutation C1483A.
