# Supplementary material for: Combination of X-ray crystallography, SAXS and DEER to obtain the structure of the FnIII-3,4 domains of integrin α6β4
Source: Acta Crystallogr D Biol Crystallogr. 2015 Mar 27;71(Pt 4):969–85. doi: 10.1107/S1399004715002485 (PMC4388270; doi:10.1107/S1399004715002485)
Supplement: Supplementary file 1 [file d-71-00969-sup1.pdf]

BIOLOGICAL CRYSTALLOGRAPHY

Volume 71 (2015)

Supporting information for article:

Combination of X-ray crystallography, SAXS and DEER to obtain the structure of the FnIII-3,4 domains of integrin α6β4

Noelia Alonso-García, Inés García-Rubio, José A. Manso, Rubén M. Buey, Hector Urien, Arnoud Sonnenberg, Gunnar Jeschke and José M. de Pereda

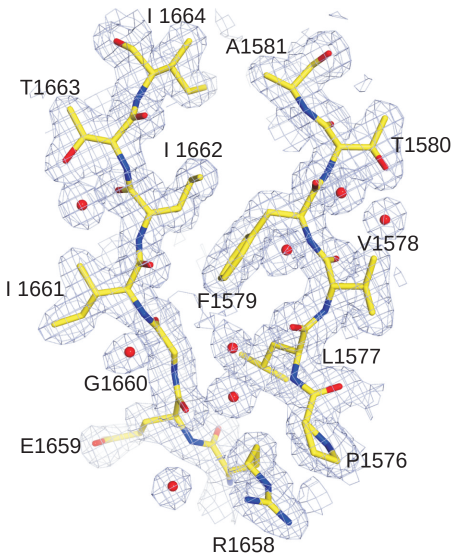

Figure S1. Stereo view of a section of an electron density map of the FnIII-4 structure (contoured at 1 ó) calculated with the phases obtained by SIRAS. The model refined against the native-1 dataset (1.80 Å resolution) is shown. This SIRAS map corresponds to the same region of the FnIII-4 structure shown in Fig 1e.

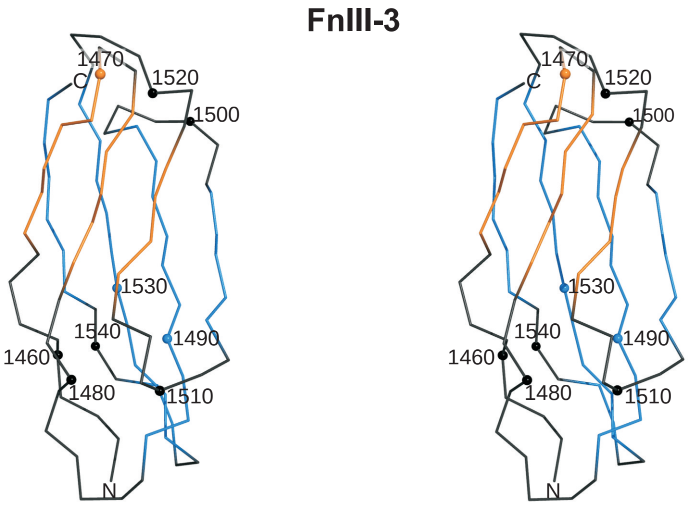

(*a*)

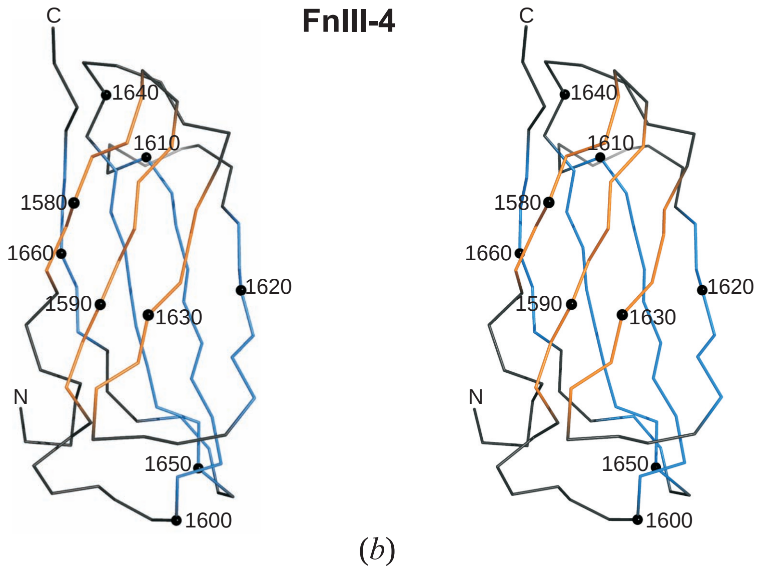

(*b*)

Figure S2. Stereo Cá-trace representation of the structures of the FnIII-3 (*a*) and the FnIII-4 (*b*). The structures are shown in the same orientation as in the left-side views in Fig. 1b-c. The position of every tenth residue is labeled.

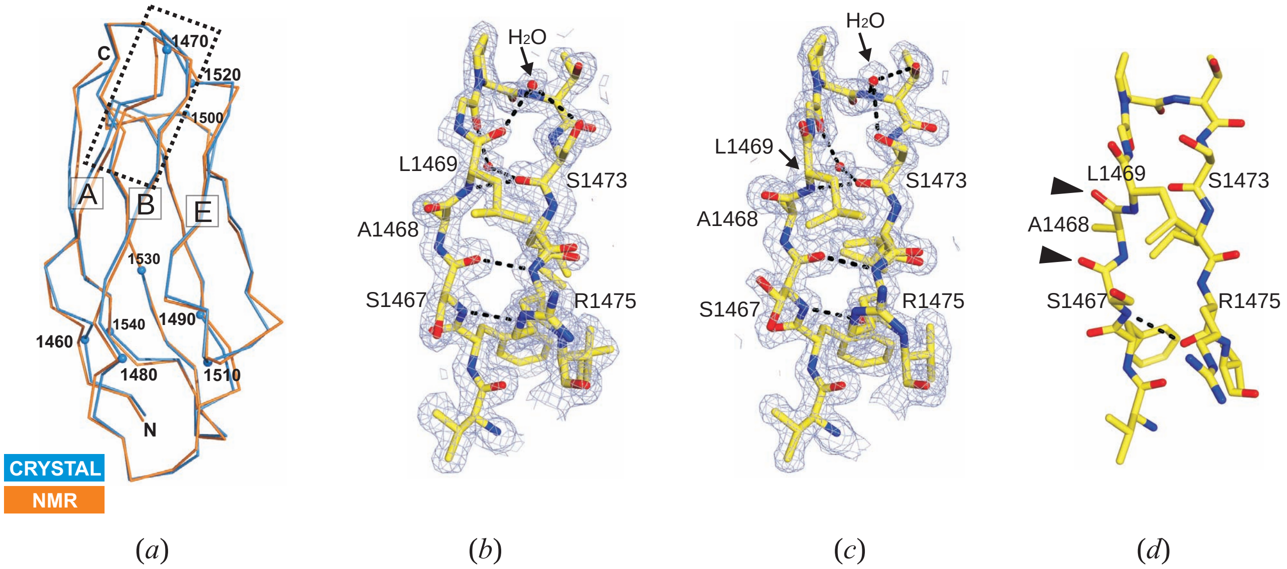

Figure S3. Comparison of the structures of the FnIII-3 domain determined by x-ray crystallography and NMR. (*a*) Cá-trace representation of the NMR structure of the FnIII-3 domain (PDB code 2YRZ) (orange) superimposed onto the crystal structure (blue). The dashed-line rectangle indicates the sections shown in detail in *b-d*. (*b*) Close-up of the region around the â-strands A-B in the first â4 molecule of the crystal structure (chain A). A 2mFobs-DFcal simulated annealing omit map, contoured at 1ó, and the refined model are shown. The map was calculated using phases from a model in which the region 1466-1475 of this molecule was removed, the B-factors were reset to 14 Å^2, and was then refined by simulated annealing (start temperature 4000 K) with Phenix Refine (Adams *et al*., 2010). Hydrogen bonds are shown as dashed lines. S1467 and L1469 make canonical â-sheet hydrogen bonds to R1475 and S1473, respectively. (*c*) Similar representation of the second molecule (chain B) in the asymmetric unit of the crystals. (*d*) Close-up of the same region in the NMR structure. The carbonyl groups of S1467 and A1468 (marked by arrowheads) are flipped with respect to the crystal structure. For clarity, only the first model of the NMR ensemble is shown; the structure of this region is highly similar in all the deposited models.

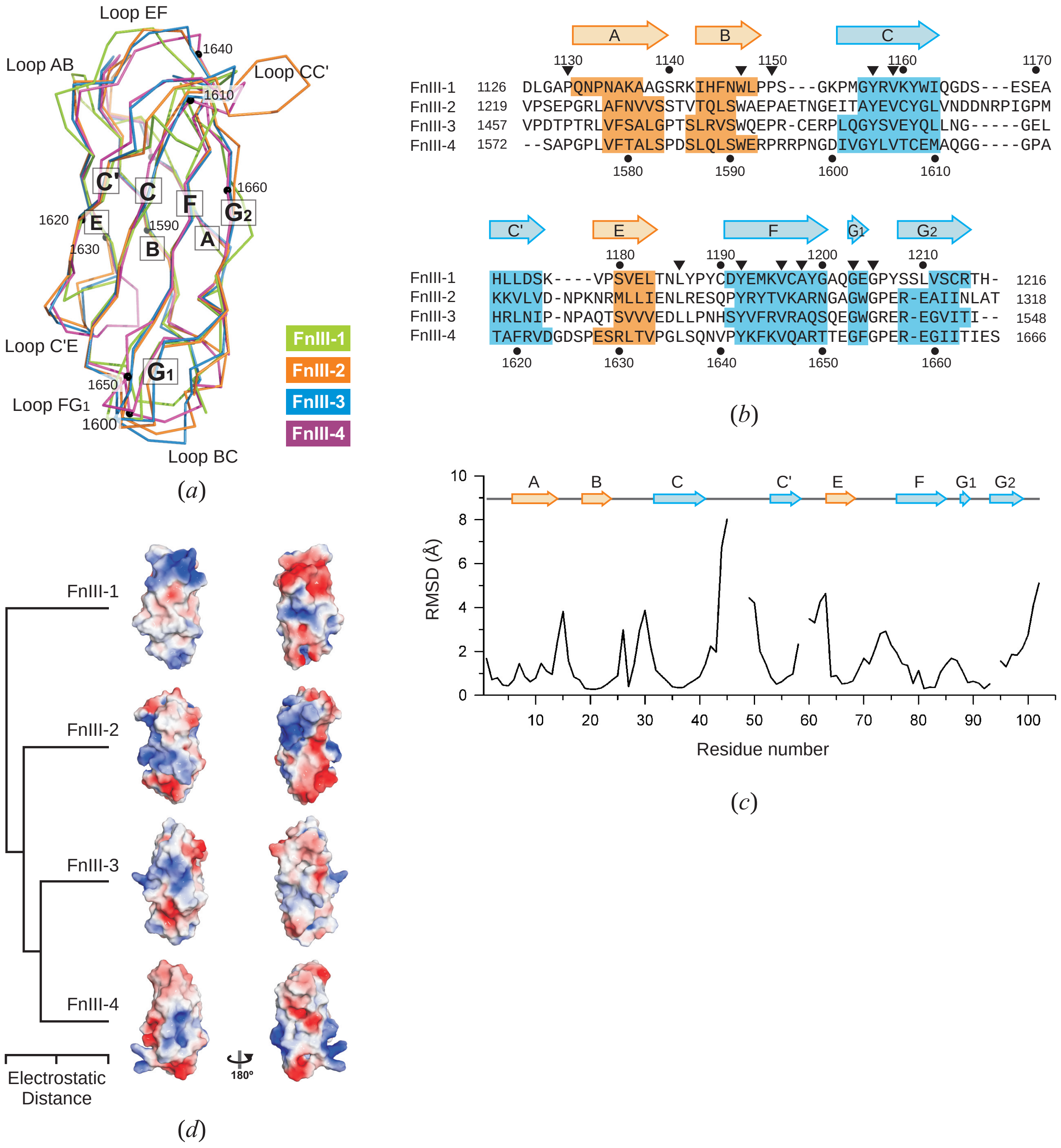

Figure S4 Structural comparison of the four FnIII domains of the integrin â4. (*a*) Cá-trace representation of the four FnIII domains of â4 after simultaneous superimposition using a maximum likelihood method with the program THESEUS (Theobald & Steindel, 2012). Every tenth residue in the FnIII-4 is marked with a sphere and is labeled. (*b*) Multiple sequence alignment based on the structural superimposition shown in *a*. The â-strands in each domain are indicated by colored boxes and the consensus extension of the secondary structure elements is shown on top. Positions with identical residues in the four domains are indicated by inverted triangles. (*c*) Representation of the r.m.s.d. of the Cá atoms of the four FnIII domains at each position of the structural alignment. Gaps in the plot correspond to positions of the alignment present in only one of the domains. (*d*) Representation of the molecular surface of the four FnIII domains

colored by the electrostatic potential, from -3 kT/e (red) to +3 kT/e (blue). In the left images the domains are oriented with the ABE â-sheet in the front, similarly to the view shown in figure 1b-c. The four FnIII domains were simultaneously superimposed and the electrostatic potentials were compared with the webPIPSA server (Richter *et al*., 2008). The domains were clustered according to the pairwise electrostatic distances estimated with PIPSA and the clustering is represented as a dendrogram on the left.

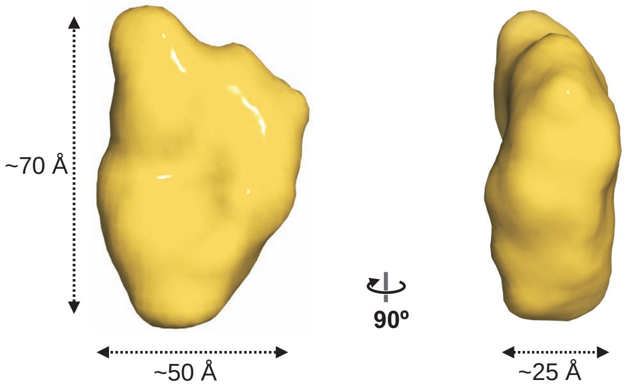

Figure S5. Reconstruction of the structure of FnIII-3,4 from SAXS data with the program DALAI_GA (Chacon *et al*., 2000). Two orthogonal views of a molecular envelope that corresponds to the average of 20 independent reconstructions.

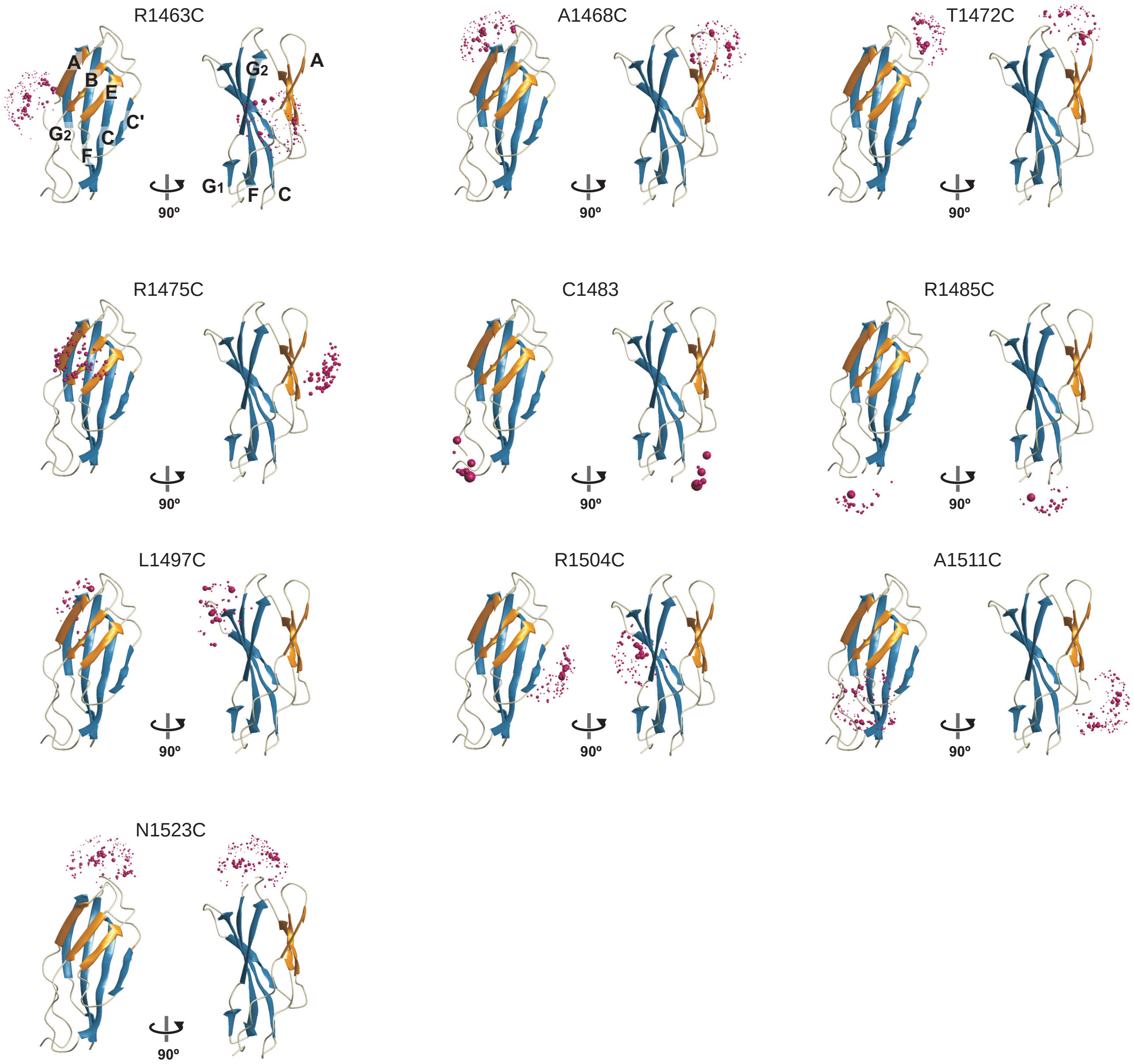

Figure S6. Modeling of the MTSL probe attached to Cys in the FnIII-3 domain. The positions of the nitroxide (N-O) paramagnetic center in the possible rotamers of MTSL attached at ten Cys of the FnIII-3 are shown as purple spheres on the crystal structure. The conformations of the probe were modeled with the MMM package (Polyhach *et al.*, 2011). The radii of the spheres are proportional to the frequency of the corresponding rotamer in the pool of simulated rotamers. Two orthogonal views are shown for each labeling position.

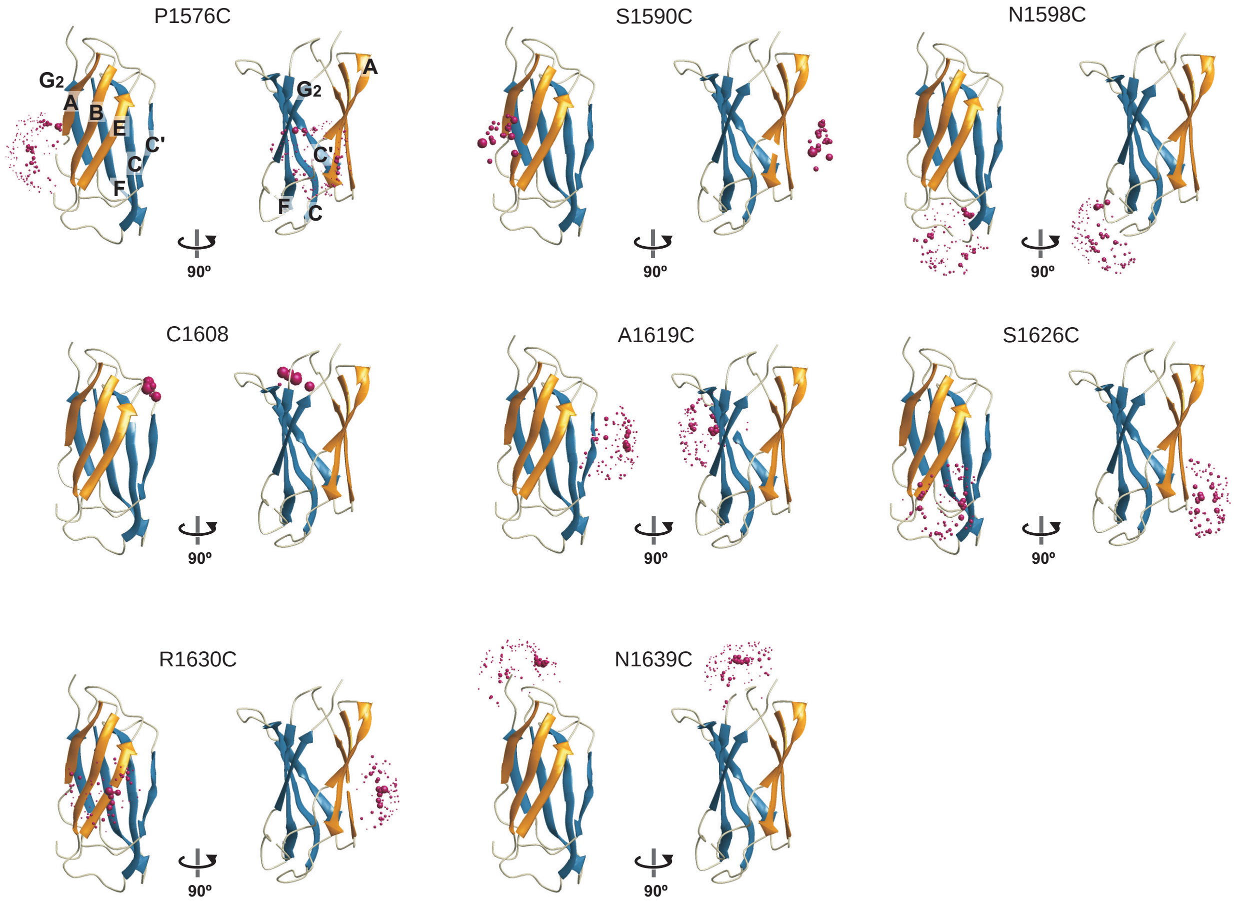

Figure S7. Modeling of the MTSL probe attached to Cys in the FnIII-4 domain. Crystal structure of the FnIII-4 with the positions of the nitroxide (N-O) paramagnetic centers of MTSL modeled attached to eight labeling positions. The radii of the spheres are proportional to the frequency of the corresponding rotamer in the pool of simulated rotamers. Two orthogonal views are shown for each labeling position.

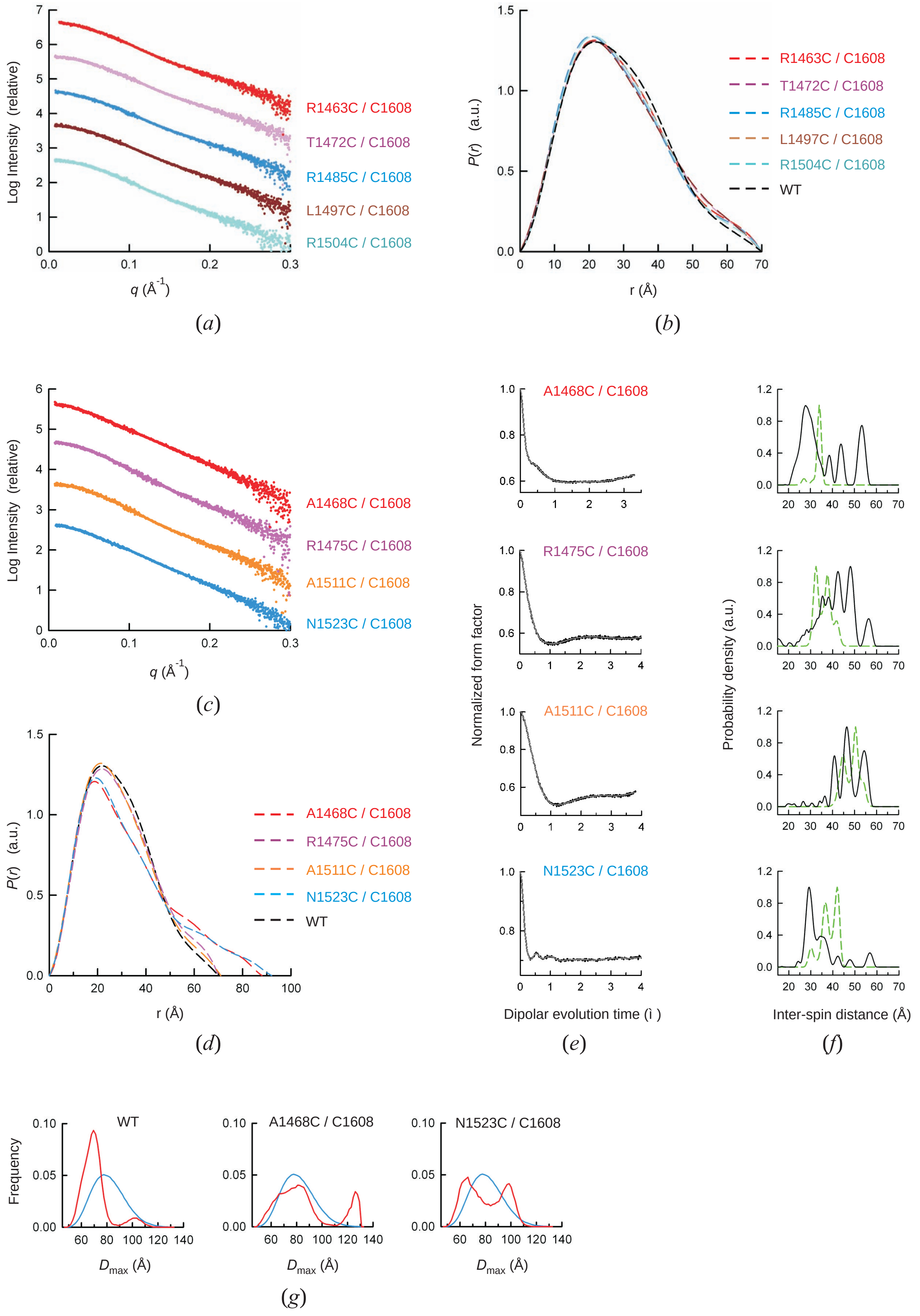

Figure S8. SAXS and DEER analysis of doubly MTSL-labeled FnIII-3,4 mutants that contain C1608 in the FnIII-4. (*a*) SAXS profiles extrapolated to infinite dilution of the MTSL-labeled FnIII-3,4 mutants

R1463C/C1608 (red), T1472C/C1608 (violet), R1485C/C1608 (blue), L1497C/C1608 (brown), and R1504C/C1608 (aquamarine). (*b*) Pair-distance distribution functions, *P*(*r*), calculated from the scattering profiles in *a* (same color coding). The *P*(*r*) calculated from the SAXS data of the unlabeled wild type FnIII-3,4 protein is shown (dashed black line) for comparison in *b* and *d*. (*c*) SAXS profiles extrapolated to infinite dilution of the FnIII-3,4 mutants A1468C/C1608 (red), R1475C/C1608 (violet), A1511C/C1608 (orange), and N1523C/C1608 (blue), labeled with MTSL. These mutants also contain the substitutions C1483S and C1559A. (*d*) *P*(*r*) functions calculated from the scattering profiles in *c*. (*e*) DEER normalized dipolar evolution (black line) and fits to the data (gray line) for the same mutants as in *c* and *d*. (*f*) Inter-spin distance distribution calculated from the data in *e* (black line) and simulated distance distribution calculated for a model of the FnIII-3,4 region (dashed green line). The model reproduces the main inter-spin distance peaks of the A1511C/C1608 mutant within 4 Å. The distribution calculated for the R1475C/C1608 mutant is skewed towards shorter distances than those observed. This could be due to a shift in the rotamer distribution of the probe due to the proximity of the inter-domain linker that is predicted to lie near R1475. Finally, differences in the experimental and calculated distance distribution of mutants A1468C/C1608 and N1523C/C1608 reflect the overall structural changes of these mutants with respect to the wild type protein. (*g*) Analysis of inter-domain flexibility of the MTSL-labeled mutants A1468C/C1608 and N1523C/C1608 using the ensemble optimization method (EOM) (Bernado *et al.*, 2007). The EOM describes the experimental SAXS profile as an averaged theoretical scattering intensity from an ensemble of conformations. A pool of 30.000 models that samples possible conformations of the FnIII-3,4 was created considering each FnIII domain as a rigid body and a flexible inter-domain. The plots represent the frequency distribution of the D_{max} in the pool (blue lines) and in the selected ensemble that fits the data (red lines). EOM analysis of the wild type FnIII-3,4 is shown for comparison. The narrow D_{max} distribution of the selected ensemble that fits the wild type data supports a compact structure. On the other hand, the wider spread of D_{max} in the selected ensembles that fit the experimental curves of A1468C/C1608 and N1523C/C1608 suggest a loose and flexible arrangement of the two FnIII domains in these mutants.

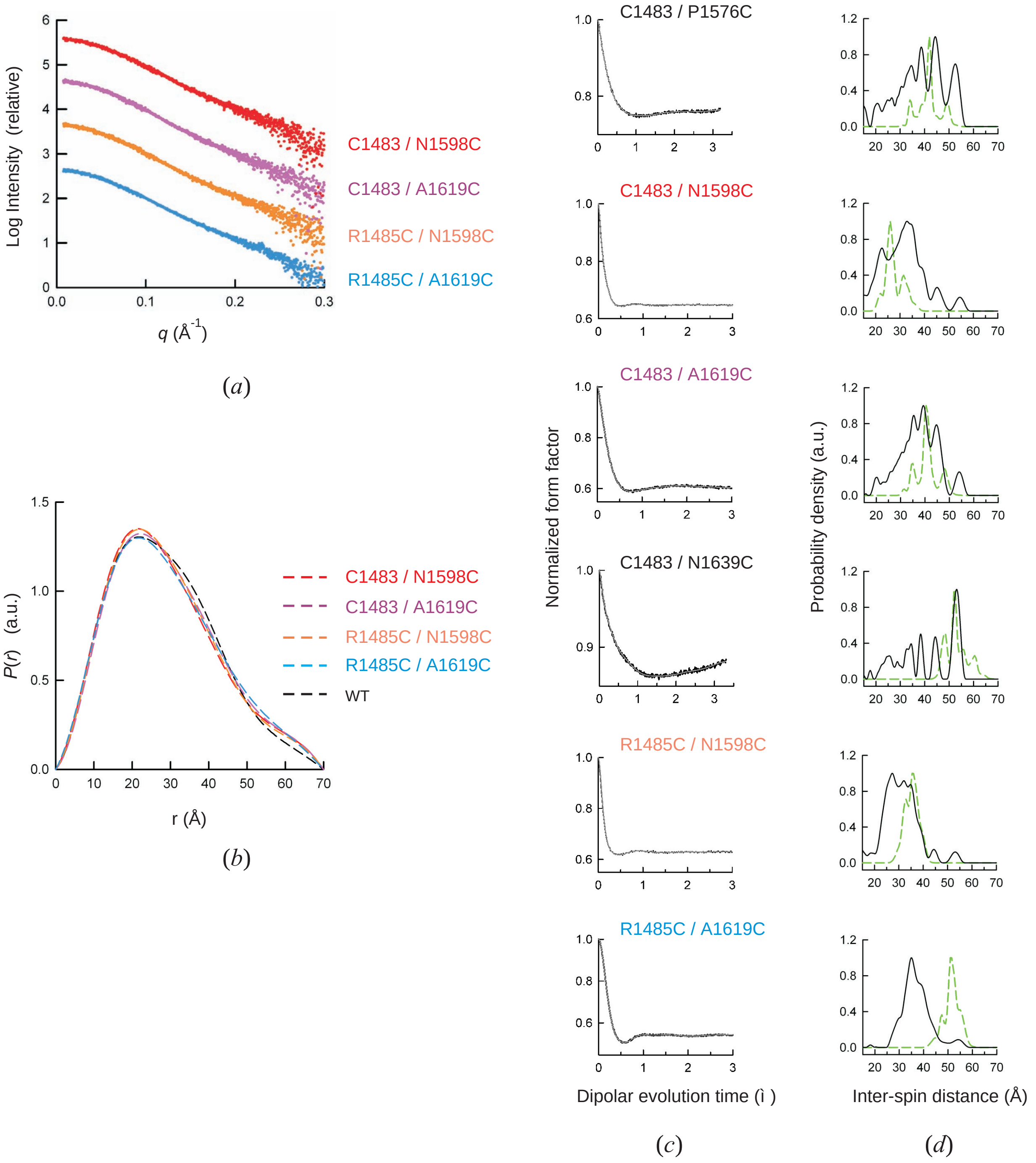

Figure S9. SAXS and DEER analysis of the doubly MTSL-labeled mutants C1483/P1576C, C1483/N1598C, C1483/A1619C, C1483/N1639C, R1485C/N1598C, and R1485C/ A1619C. (*a*) SAXS profile extrapolated to infinite dilution of the MTSL-labeled FnIII-3,4 mutants C1483/N1598C (red), C1483/A1619C (violet), R1485C/N1598C (orange), and R1485C/A1619C (blue). These mutants contain the substitutions C1559A and C1608A, and the last two also contain C1843A. It was not possible to measure SAXS data of the mutants C1483/P1576C and C1483/N1639C. (*b*) Pair-distance distribution functions calculated from the scattering profiles in *a* (curves colored as in *a*) and SAXS-derived *P*(*r*) of the

unlabeled wild type FnIII-3,4 protein (dashed black line). These mutants labeled with MTSL have similar structural parameters to those of the wild type protein. (*c*) DEER normalized dipolar evolution (black line) and fits to the data (gray line) of the mutants in *b*, C1483/P1576C, and C1483/N1639C. (*d*) Inter-spin distance distribution calculated from the data in *c* (black line) and simulated distance distribution calculated for a model of the FnIII-3,4 region (dashed green line). The simulated distributions of the mutants C1483/P1576C, C1483/N1598C, C1483/A1619C, C1483/N1639C, and R1485C/N1598C, show overall agreement with those determined by EPR. The wide distance distributions observed by EPR could reflect local distortion of the structure due to the MTSL-labeling of C1483. Only the distance distribution simulation for the mutant R1485C/A1619C showed a notable discrepancy with the experimental distribution determined by DEER.

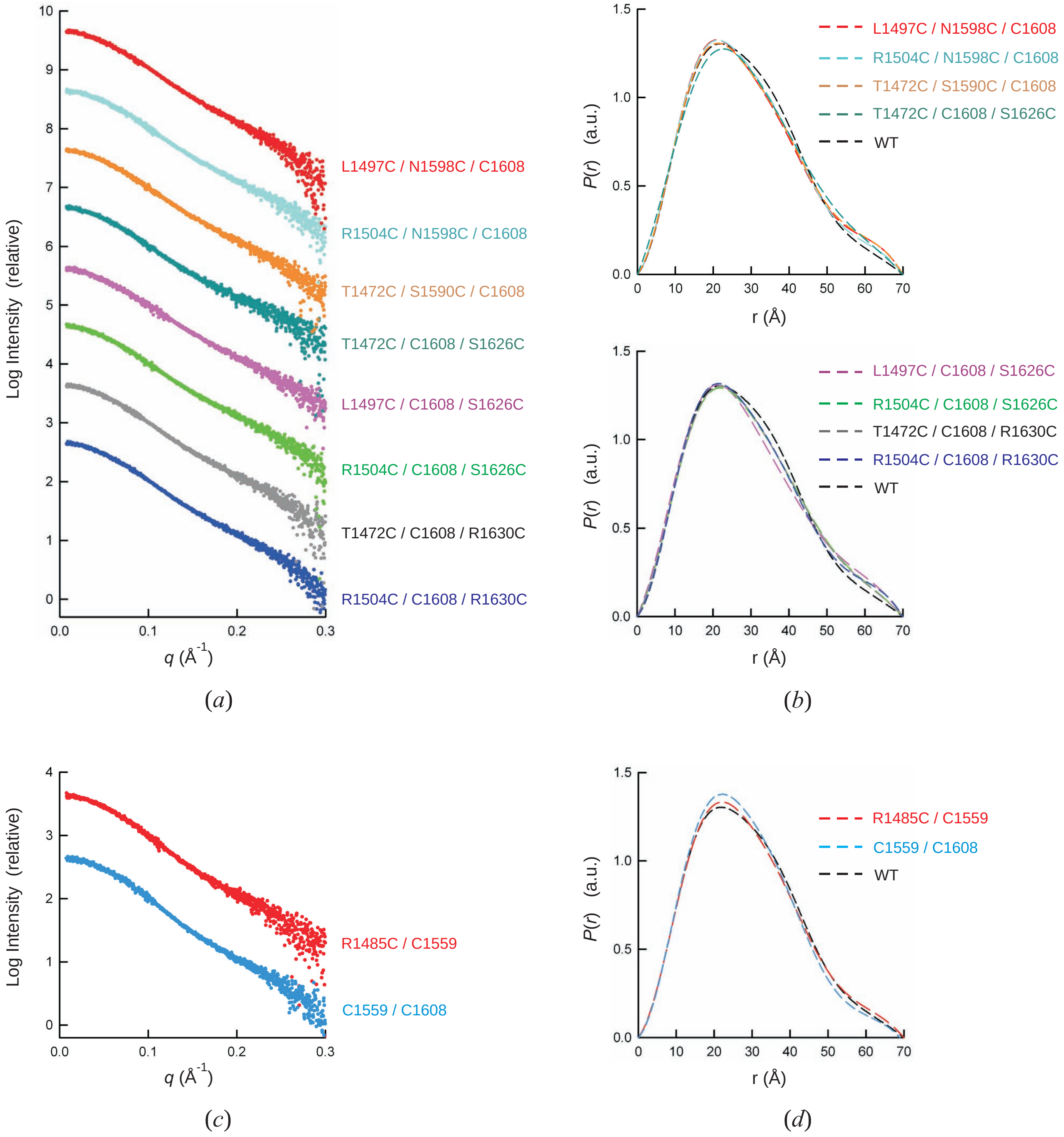

Figure S10. SAXS analysis of triply MTSL-labeled mutants of the FnIII-3,4, and doubly labeled mutants that contain C1559 in the inter-domain linker. (*a*) SAXS profiles extrapolated to infinite dilution of the MTSL-labeled FnIII-3,4 mutants (in descending order) L1497C/N1598C/C1608, R1504C/N1598C/C1608, T1472C/S1590C/C1608, T1472C/C1608/S1626C, L1497C/C1608/S1626C, R1504C/C1608/S1626C, T1472C/C1608/R1630C, and R1504C/C1608/R1630C. (*b*) *P*(*r*) functions calculated from the scattering profiles in *a*. The curves, colored as in *a*, are shown in two panels for clarity. (*c*) SAXS profiles extrapolated to infinite dilution of the FnIII-3,4 mutants R1485C/C1559 (red) and C1559/C1608 (blue), labeled with MTSL. (*d*) *P*(*r*) functions calculated from the scattering profiles in *c*. The *P*(*r*) of the unlabeled wild type FnIII-3,4 protein (dashed black line) is shown for comparison in *b* and *d*.

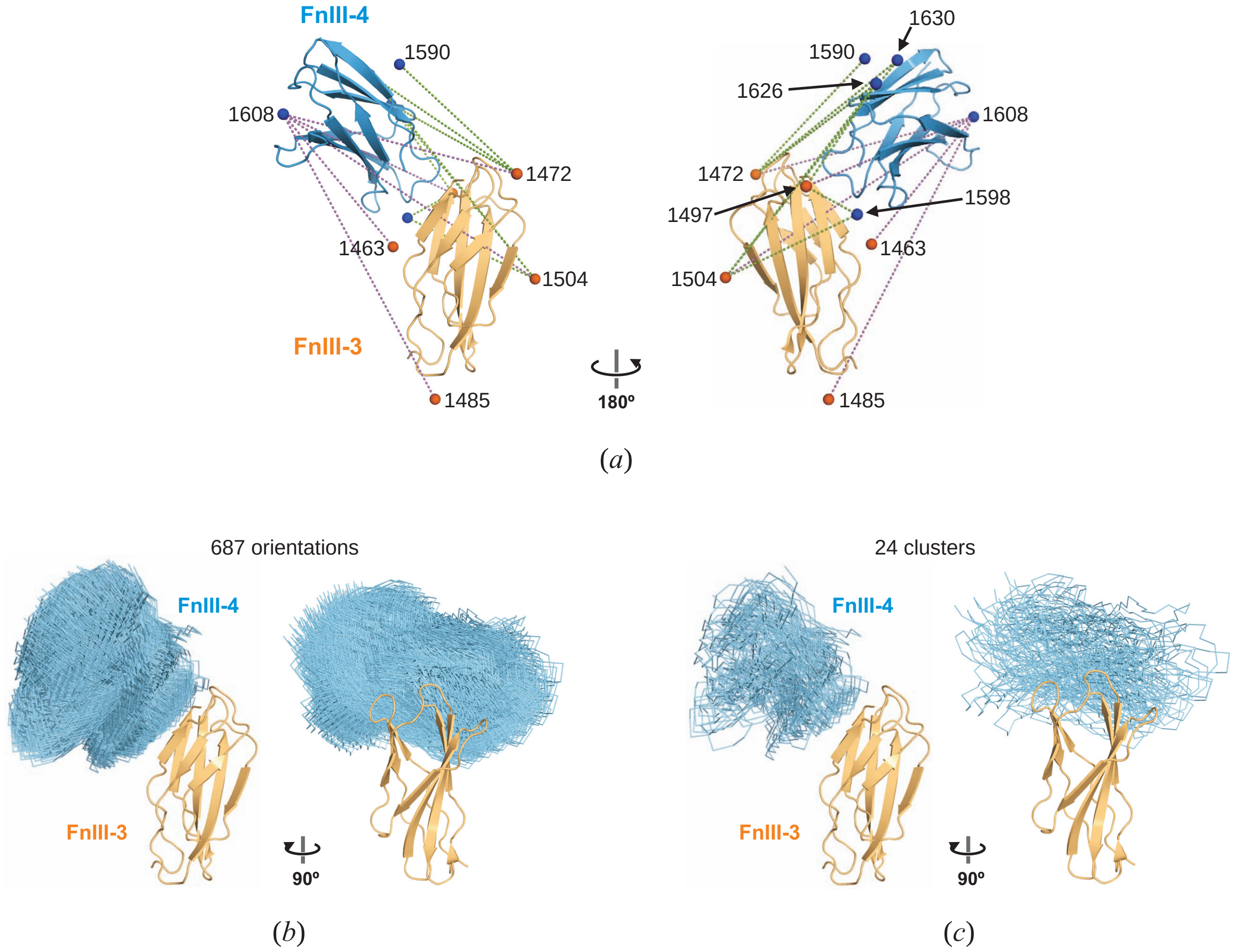

Figure S11. Determination of the relative orientation of the FnIII-3 and FnIII-4 domains. (*a*) 3-D representation of the 13 DEER distances used to model the position of the two FnIII domains. The calculated average positions of the paramagnetic group of the MTSL attached to the FnIII-3 (orange spheres) and the FnIII-4 (blue spheres) are shown in one of the final models of the FnIII-3,4 region. Distances derived from doubly labeled mutants are shown as violet dashed lines, while those obtained from triply labeled samples are indicated by green dashed lines. The wide spatial distribution of the DEER distances reduced the uncertainty in the rigid-body fitting of the two domains. (*b*) Representation of the 687 orientations of the FnIII-4 obtained by exhaustive 6-D search, which have a ó$_{\mathrm{DEER}} \leq 3$ Å for the 13 inter-domain distances shown in *a*. (*c*) Representative orientations of the FnIII-4 in the 24 groups identified by cluster analysis of the initial 687 models.

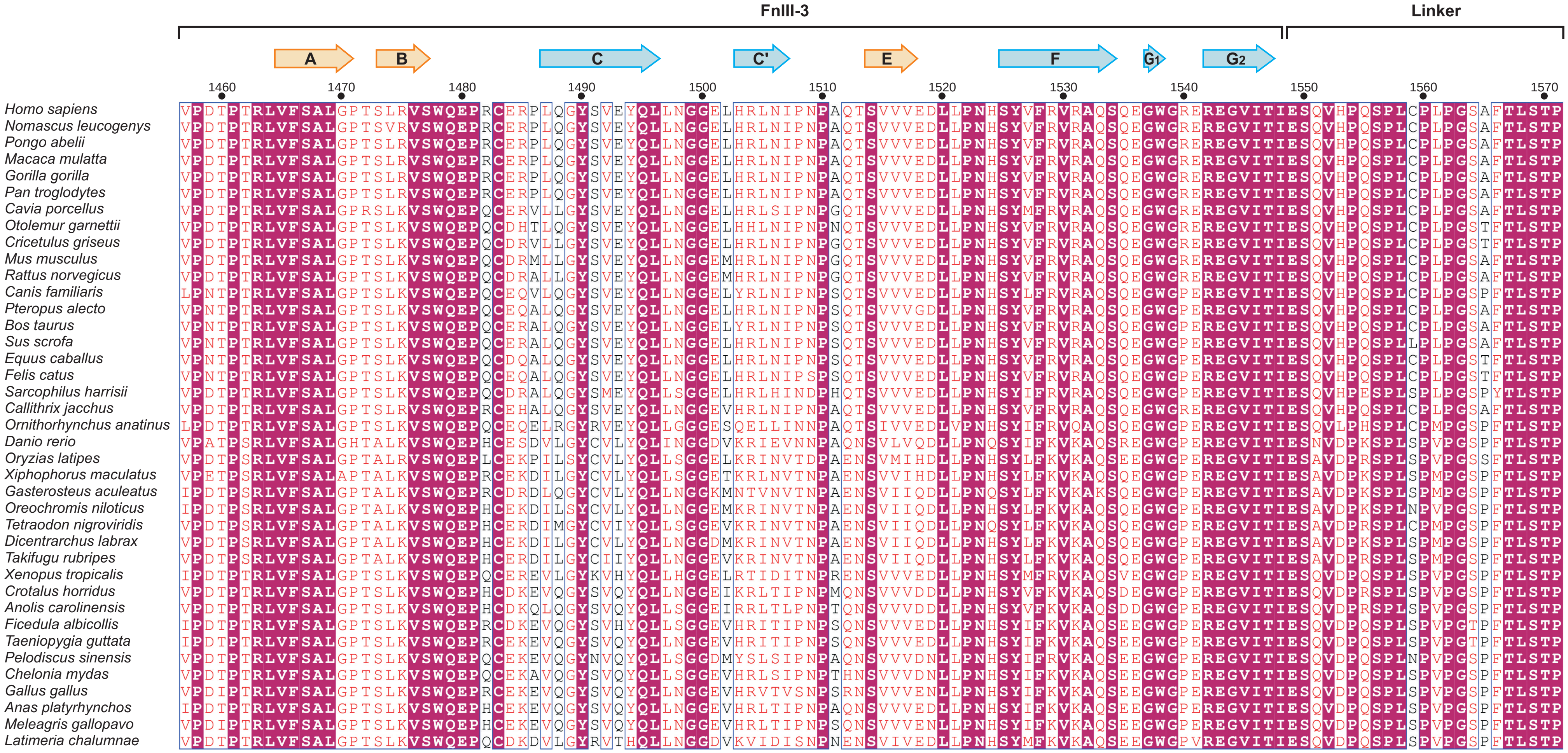

Figure S12. Multiple sequence alignment of integrin â4 sequences used to calculate the evolutionary conservation scores. The sequences of the FnIII-3,4 region from 39 species were aligned with Clustal Omega (Sievers *et al*., 2011) and the alignment was colored using the program ESPript (Robert & Gouet, 2014). Fully conserved positions are highlighted by purple boxes. The secondary structure elements in the structures of the human FnIII-3 and FnIII-4 domains are indicated on top.

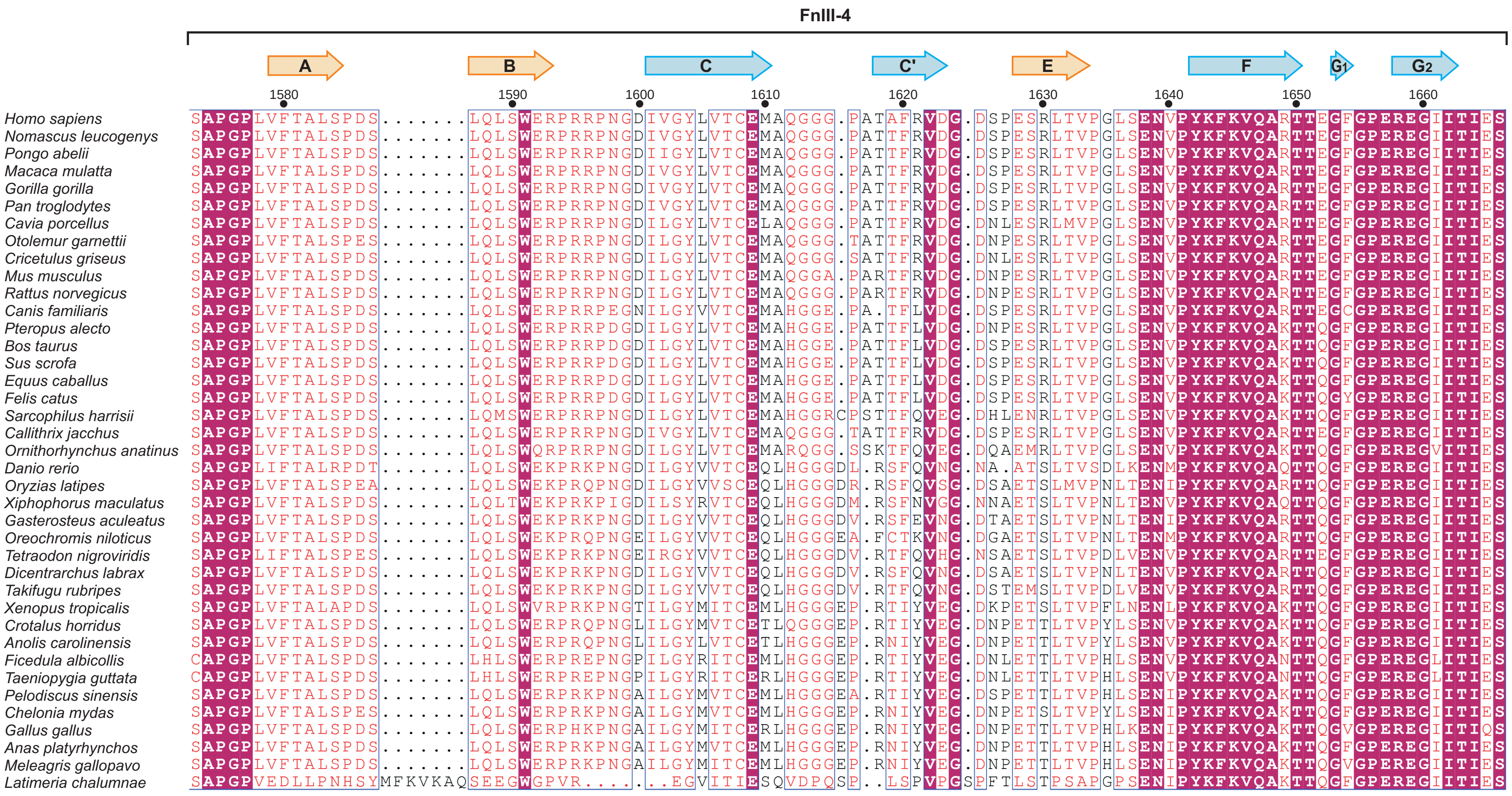

Figure S12. (continuation)

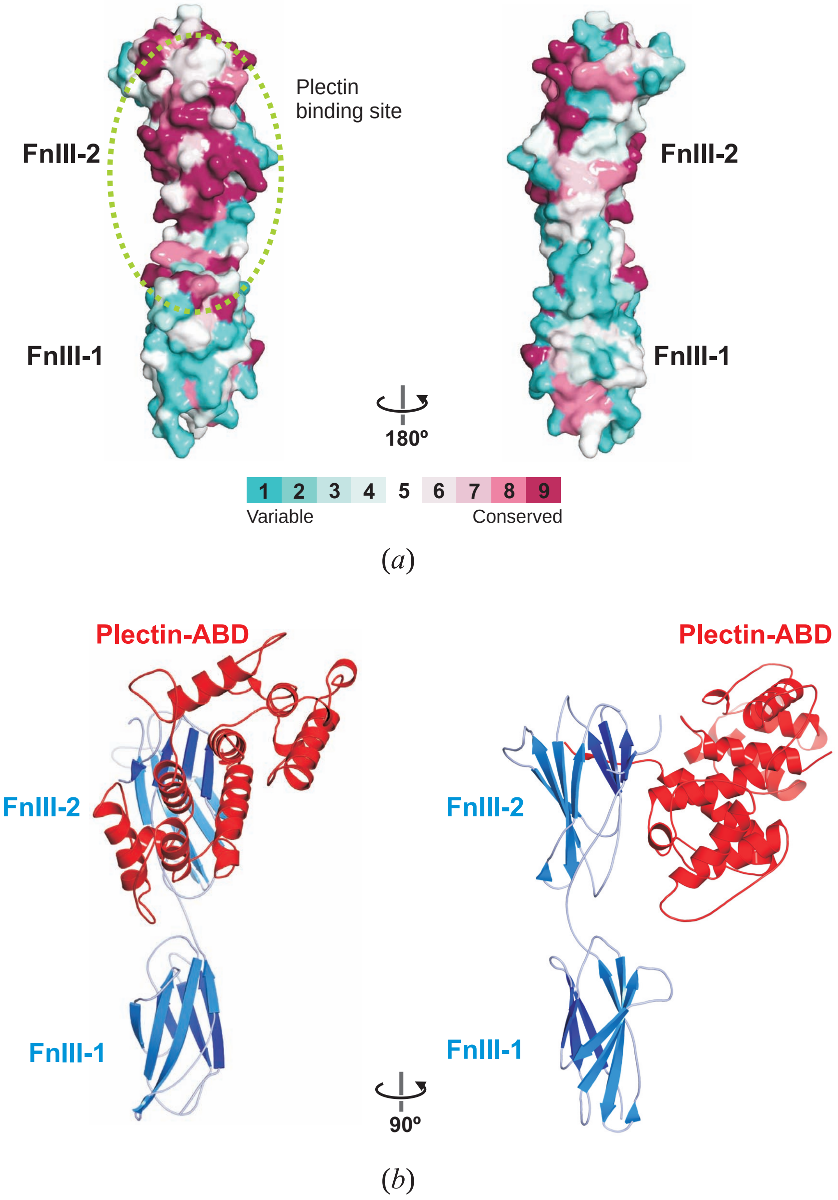

Figure S13. Evolutionary conservation of the plectin-binding site in the FnIII-1,2 of â4. (*a*) Surface representation of the structure of the FnIII-1,2 (PDB code 1QG3) (de Pereda *et al*., 1999) colored by the evolutionary conservation grade of the amino-acids in a set of â4 sequences of 39 species; calculated with the ConSurf server. The plectin binding site (dashed line oval) is highly conserved, while other surface areas are mostly variable. (*b*) To illustrate the interaction of the FnIII-1,2 with plectin, the structure of the FnIII-1,2 of â4 (blue) bound to the ABD of plectin (red) (PDB code 3F7P) (de Pereda *et al*. 2009a) is shown. In the image on the left side, the FnIII-1,2 is shown in a similar orientation as in the left-side representation in *a*.

SUPPLEMENTARY TABLE

Table S1. Additional FnIII-3,4 Cys mutants and structural parameters estimated from SAXS analysis

FnIII-3,4 mutant	Spin labeled sites		Structural parameters [d]	
	FnIII-3	FnIII-4	R_g (Å)	D_{max} (Å)
A1468C / C1608 [a]	1468	1608	26.1	88
R1475C / C1608 [a]	1475	1608	23.0	70
R1511C / C1608 [a]	1511	1608	22.4	70
N1523C / C1608 [a]	1523	1608	26.1	92
C1483 / P1576C [b]	1483	1576	n.d.	n.d.
C1483 / N1598C [b]	1483	1598	22.4	70
C1483 / A1619C [b]	1483	1619	22.7	70
C1483 / N1639C [b]	1483	1639	n.d.[e]	n.d.
R1485C / N1598C [c]	1485	1598	22.4	70
R1485C / A1619C [c]	1485	1619	22.8	70

[a], proteins contain the C1483S and C1559A substitutions.
[b], proteins contain the C1559A and C1608A substitutions.
[c], proteins contain the C1483A, C1559A, and C1608A substitutions.
[d], determined from the SAXS profile by indirect-transform using data for $q \leq 0.3$ Å^{-1}.
[e], not determined.
